# Advances of medical nanorobots for future cancer treatments

**DOI:** 10.1186/s13045-023-01463-z

**Published:** 2023-07-14

**Authors:** Xiangyi Kong, Peng Gao, Jing Wang, Yi Fang, Kuo Chu Hwang

**Affiliations:** 1 Department of Breast Surgical Oncology, National Cancer Center/National Clinical Research Center for Cancer/Cancer Hospital, Chinese Academy of Medical Sciences and Peking Union Medical College, Beijing, 100021 China; 2 Department of Breast Surgical Oncology, National Cancer Center/National Clinical Research Center for Cancer/Cancer Hospital and Shenzhen Hospital, Chinese Academy of Medical Sciences and Peking Union Medical College, Shenzhen, 518116 China; 3 Division of Breast Surgery, Department of General Surgery, West China Hospital, Sichuan University, Chengdu, 610041 China; 4 Breast Center, West China Hospital, Sichuan University, Chengdu, 610041 China; 5 Department of Chemistry, National Tsing Hua University, Hsinchu, 30013 Taiwan ROC

**Keywords:** Nanorobots, Cancer treatment, Drug delivery, Targeted therapy

## Abstract

Early detection and diagnosis of many cancers is very challenging. Late stage detection of a cancer always leads to high mortality rates. It is imperative to develop novel and more sensitive and effective diagnosis and therapeutic methods for cancer treatments. The development of new cancer treatments has become a crucial aspect of medical advancements. Nanobots, as one of the most promising applications of nanomedicines, are at the forefront of multidisciplinary research. With the progress of nanotechnology, nanobots enable the assembly and deployment of functional molecular/nanosized machines and are increasingly being utilized in cancer diagnosis and therapeutic treatment. In recent years, various practical applications of nanobots for cancer treatments have transitioned from theory to practice, from in vitro experiments to in vivo applications. In this paper, we review and analyze the recent advancements of nanobots in cancer treatments, with a particular emphasis on their key fundamental features and their applications in drug delivery, tumor sensing and diagnosis, targeted therapy, minimally invasive surgery, and other comprehensive treatments. At the same time, we discuss the challenges and the potential research opportunities for nanobots in revolutionizing cancer treatments. In the future, medical nanobots are expected to become more sophisticated and capable of performing multiple medical functions and tasks, ultimately becoming true nanosubmarines in the bloodstream.

**Graphical abstract:**

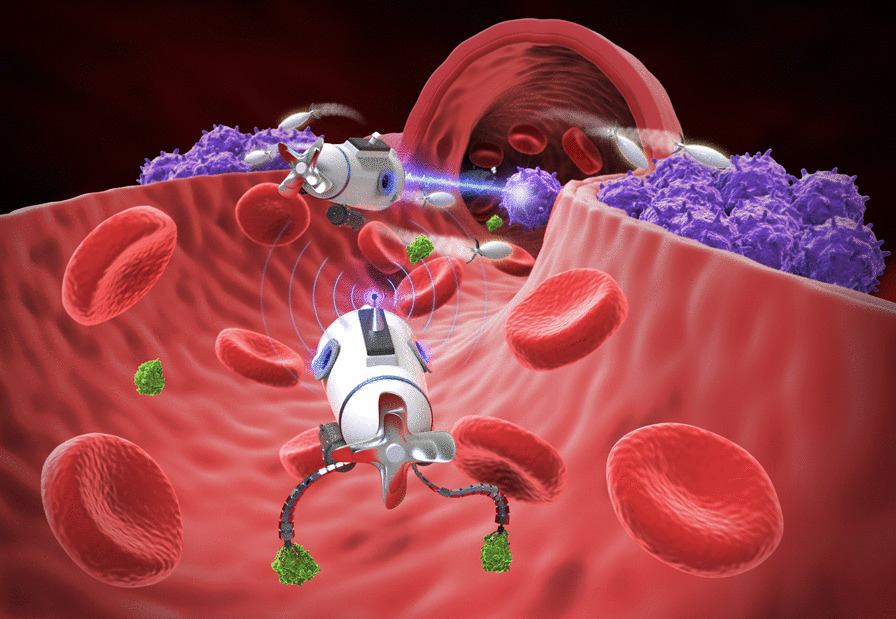

**Supplementary Information:**

The online version contains supplementary material available at 10.1186/s13045-023-01463-z.

## Introduction

**Fig. 1:**
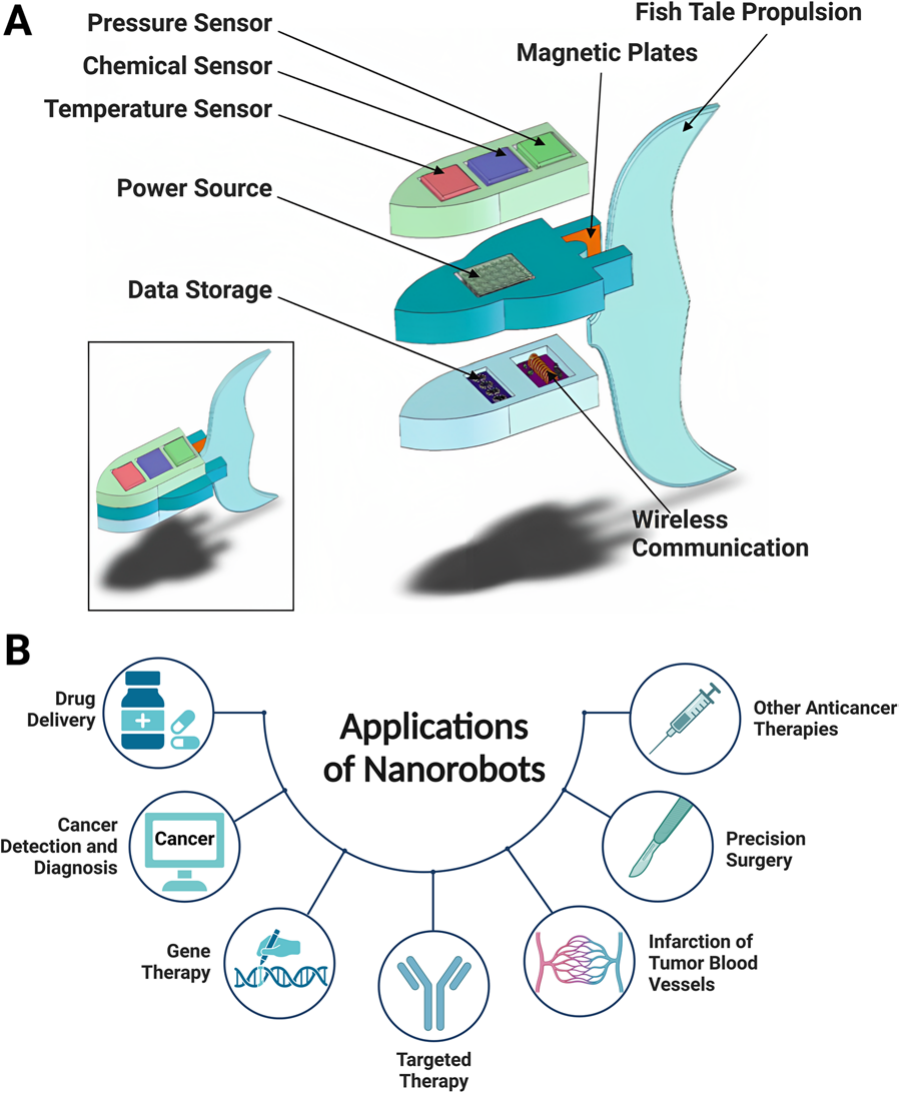
Core structure of nanorobots with their components and applications. **A** Detailed view of a fully functional, autonomous nanorobot for cancer treatment and its individual components. Modified and reprinted from ref [8]. with permission, Copyright 2013, Springer Science Business Media, LLC. **B** Illustration of the potential applications of nanorobots in combating cancers. Created with BioRender.com. The “confirmation of publication and licensing rights” was shown in Additional file 1

Using nanomolecular scale tools and biological nanomolecular knowledge of the human body, nanomedicines were designed to aim at treating and preventing diseases, preserving and improving human health [1, 2]. With the great development potential and application prospects in the treatment of tumors, the development of nanomedicines was very rapid in the last few decades [3, 4]. Nanorobots, as one of the most promising applications of nanomedicines, allow one to access remote and hard-to-reach body regions, and perform various medical tasks [5-7] (Fig. 1).

Medical nanorobots are defined as untethered nanostructures that contain an engine or are capable of transforming diverse types of energy sources to mechanical forces and perform a medical task [9-14]. Due to their small sizes, nanorobots can directly interact with cells and even penetrate them, providing direct access to the cellular machineries [15, 16]. As an interdisciplinary technology, nanorobots address the assembly and utilization of functional nano-to-molecular scale machines and have been widely used in cancer diagnosis and treatment. Nanorobots are nanosized machineries able to deliver payloads (drugs, genes, sensing molecules, etc.), achieve some certain (biomedical) functions (diagnosis, therapeutic actions), have targeting ability to search for tumor/disease sites, as well have an active or passive power system able to receive external power sources (NIR light, ultrasound, magnetic driving force, etc.) or to utilize the mediums/blood flow existing in a biological system. The key differences between nanorobots and nanocarriers are the active power system. Nanomedicines/nanocarriers can also be considered or included as a part of nanorobots, but without having an active power system. Researchers worldwide have devoted themselves to the research and development of cancer-killing nanorobots in the hope to introduce them into clinical practices and to accomplish a medical modernization. One of the unmet and major challenges of nanorobotic technology is to introduce these nanorobotic tools to real-world clinical practices.

In recent years, various practical applications of micro- and nanorobots for cancer treatments have been realized from theory to practice, from in vitro experiments to in vivo applications. The size of a single biomolecule is at the nanometer scale, which limits the operation of microrobots. Robotic manipulations of biomolecules require the use of nanorobots with the same or a similar nanoscale [17-20]. In the process of exploring nanotechnology from laboratory to clinics for cancer treatment, nanorobots can achieve a variety of medical functions, including drug delivery, tumor detection and diagnosis, targeted therapy, minimally invasive surgery, and other comprehensive tasks [21-28]. Miniaturization of the robotic technology and its combination with advanced medical technologies make it possible for numerous biomedical applications, including precision and targeted medication. Each of these applications aims to address and conquer different challenges in the treatments of cancers.

Although a few previous reviews have covered generalized or specific topics for the use of micro- and nanorobots in medicine [11, 16, 21, 29, 30], most reviews address the biomedical applications of microrobots with no reviews focusing on the recent efforts of nanorobots in the treatments of cancers. After a brief overview of natural biological nanomachines and the key fundamentals of nanorobots, this review provides a comprehensive overview of the recent advances of nanorobots from the perspective of cancer treatments, mainly focusing on the biomedical applications of nanorobots, and highlights the most promising research opportunities that may have profound impacts on cancer treatment in the next few decades. In the future, medical nanorobots will be developed to become much more sophisticated and are able to perform multi-medical functions and tasks, and eventually become true nanosubmarines in the blood.

## Natural nanorobots existing in biological systems

We have witnessed great advances in nanotechnology in the past decades, and a large number of novel nanotechnologies have been discovered and applied to a wide range of fields. However, living organisms present us with some impressive natural nanomachines which can be viewed as “bionanorobots” [31, 32]. These natural nanorobots that can both rotate and transport chemical loads following predetermined tracks with subnanometer precision and high efficiency are essential for a plethora of cellular functions [33]. Numerous natural biological nanorobots are found to use energy to do their assigned work and transform it into mechanical work in living systems.

Most protein-based molecular nanorobots can convert the chemical energy ATP (adenosine triphosphate) into mechanical motion [34]. As one of the most prevalent and abundant proteins on Earth, ATP synthase is not only found in the thylakoid membrane of chloroplasts, the cristae of mitochondria, but also in the plasma membrane of bacteria [35]. As the last enzyme in the oxidative phosphorylation pathway, this natural nanomachinery is able to use electrochemical energy to power the synthesis of ATP, and it converts ADP and phosphate into ATP molecules, which self-rotate due to the electrochemical gradient and the flow of protons through the membrane [33]. ATP synthase enzyme is vital to the well-being of humans and has the potential to contribute to new approaches to cancer, bacterial infections, and obesity [36-38].p-glycoprotein (p-gp) is an ATP-binding cassette transporter that can endow multidrug resistance against chemodrugs, notably to anticancer agents [39]. As an efflux pump that has ATPase-like function to export chemodrugs out of cells, p-gp is overexpressed in tumor cells, and drugs can be delivered to the extracellular matrix with these pumps [40]. P-gp, a 170-kD protein containing two amino acid chains, has a flexible structure capable of maintaining its rotational and translational motion in the efflux mechanism. Inhibition of this efflux activity has been one of the many aims in the exploitation of p-glycoprotein inhibitors. Many studies reported the p-gp inhibitors based on the information on the function and structure of p-glycoprotein [41, 42].

As numbers of cytoskeletal nanomotor protein families, kinesins and myosins can accomplish the corresponding work by actively transporting molecules or moving proteins within the cell. Kinesins and myosins are multi-protein complexes with motor domains, which have the ability to mediate the interaction between motor and track with high precision. The motor structural domain is chemically and functionally linked to an extended tail that is responsible for mediating the binding of cargo to the motor. And the wealth of motor structures is the key for them to produce force by undergoing similar rearrangements [43-45].

CRISPR (clustered regularly interspaced short palindromic repeats) and its related coding genes constitute the CRISPR-Cas system, which is currently known to be the only one to acquire immune system in prokaryotes [46]. As a large multi-domain and multifunctional DNA (deoxyribonucleic acid) nucleic acid endonuclease, Cas9 is the signature protein of the type II CRISPR-Cas system. Similar to natural nanorobots, Cas9 nuclease enzymes firstly search for PAM (protospacer adjacent motif) sequences on the target gene to select a target for cutting, then design an sgRNA (single-guide RNA) sequence complementary to the targeted gene, and then use the Cas9-sgRNA complex to cut the target gene to produce a DNA double-strand break, which triggers a base mismatch to achieve gene knockdown [47, 48]. CRISPR-Cas9 knockdown is more efficient and more specific than RNAi (RNA interference) gene silencing. Moreover, CRISPR-Cas9 is currently used for high-throughput genetic screening [49]. The success of in vitro play purification of Cas9 has allowed scientists to maturely use CRISPR-Cas9 knockout technology to study the function of specific genes, opening a new chapter in molecular biology.

Recent advances in techniques to investigate the mechanisms and molecular structure present a wealth of information that allows us to gain a better understanding the differences and similarities of these nanomotor systems. Many such natural biological nanorobots are found and operate in living systems that could be used for therapeutic purposes. Inspired by natural nanorobots, numerous scientists have recently researched on artificial nanomachineries in order to emulate these bionanorobots and tackle the problem of cancer treatment at the nanoscopic level. The next step was to develop systems with key fundamentals that are able to move autonomously and precisely when nanorobots perform specific work in the human body environments [50].

## Key fundamentals of nanorobots in the treatment of cancers

As a miniature structure, nanorobots are capable of executing predetermined missions and bear stark differences to their macroscale robotic counterparts. The primary challenges in the development of nanorobots or nanomechanical components lie in their construction and control. These devices operate within a microenvironment that exhibits physical characteristics distinct from those encountered by conventional components. The composition and structure of nanorobots are not uniform and can vary depending on their intended function and the materials and technologies utilized in their creation. The field of nanorobotics is an ever-evolving one, with ongoing advancements and breakthroughs. In this regard, we have presented a general outline of some of the crucial components and structures commonly found in nanorobots (Table 1) and provided a summarization of typical examples of medical nanorobots (Table 2), based on the study by Suhail et al. [51]. Currently, most nanorobot experiments are conducted under conditions akin to those found in human microenvironments. To ensure that nanorobots can effectively eliminate cancer cells within the human body, scientists have set stringent standards for their fundamental design elements. It is noteworthy that medical nanorobots are still in the nascent stages of development and are yet to be widely implemented in medical treatments. The specific composition and structure of these devices may greatly vary based on their intended application and the necessary requirements for safety, efficacy, and scalability.

**Table 1:** Overview of some key components and their structures of medical nanorobots

Entry	Component	Description	Structure
1	Shell	The exterior casing of the medical nanorobot, which is designed to be biocompatible with the human body. The material used for the shell can vary, but common options include silicon, carbon, and diamond	The structure of the shell plays a crucial role in the performance and safety of a nanorobot. For instance, a smooth and spherical shell design reduces friction and decreases the likelihood of causing damages to surrounding tissues. On the other hand, a rough and irregular shell design can enhance the nanorobot’s ability to attach to target at cancer cells or tumor tissues. The shape, size, and surface features of the shell can be optimized for specific applications
2	Power source	Medical nanorobots need an energy source to function. This can be in the form of a battery, hydrogen fuel cell, or even energy derived from the body’s metabolism	The integration of the power source into the nanorobot’s design can greatly impact its performance, stability, and safety. The power source can be embedded within the shell or attached to the surface as an external component. The size and placement of the power source must be considered to ensure optimal functionality
6	Payload	This refers to the specific function that a nanorobot was designed to perform, such as targeted drug delivery, imaging, or tissue repair	The payload of a medical nanorobot can be integrated within its shell or attached to its surface as an external component. The type and the amounts of payloads required will depend on the intended application and the requirements for efficacy and safety. For instance, a nanorobot designed for drug delivery may have a payload of drugs or therapeutic agents, while a nanorobot designed for imaging may have a payload of imaging agents or contrast agents
3	Sensors	These are devices that allow a nanorobot to detect changes in the body, such as temperature, pH, or the presence of specific molecules	Sensors can be placed on the surface of a nanorobot or integrated within its shell. The type and the number of sensors required will depend on the intended application and the information necessary for effective operation. For instance, a nanorobot designed for imaging may have sensors to detect light or local oxygen concentrations, while a nanorobot designed for drug delivery may have sensors to detect specific biochemical signals, such as pH, GSH concentrations, etc.
4	Actuators	These are devices that enable the nanorobot to physically interact with the body, such as moving through the bloodstream, releasing drugs, or performing surgery	Actuators can be placed on the surface of a nanorobot or integrated within its shell. The type and number of actuators required will depend on the intended application and the actions necessary for effective operation. For instance, a nanorobot designed for drug delivery may have actuators to release drugs in response to specific local signals, while a nanorobot designed for surgery may have actuators to manipulate tissues or remove debris
5	Communications	Medical nanorobots may need to communicate with each other or with external devices, such as an antenna of a computer or a remote control system	Communications can be achieved through various means, such as wireless signals, electromagnetic wave signals, or physical connections. The type and range of communication required will depend on the intended application and the requirements for coordination and control. For instance, a nanorobot designed for imaging may communicate with a computer to transmit images, while a nanorobot designed for drug delivery may communicate with other nanorobots to coordinate the release of payload drugs

**Table 2:** Some typical examples of medical nanorobots

Entry	Nanorobot types	Key characteristics	Refs.
1	Pharmacyte	A medical nanorobot with a diameter of 1–2 μm Molecular indicators or chemotactic sensors are used to ensure the precision of the targeting system They can be eliminated or recuperated through centrifuge nanapheresis after finishing the tasks	[52]
2	Microchips	Nanorobots possess microchips which can conduct current signals once the molecules detect a disease The benefits are the small charge to yield and simple to operate	[53]
3	Respirocyte	A type of nanorobot that carries oxygen like an artificial red blood cell The power is achieved through endogenous serum glucose	[54]
4	Microbivores	The nanorobot is flat and spheroidal in shape for nanomedical uses With a diameter of 3.4 μm along its main axis and a diameter of 2.0 μm on the minor axis It has the phagocytic capability which is almost 80-fold higher proficient than other macrophages	[55]
5	Clottocytes	They have the ‘instant’ hemostasis biological activity which is called artificial mechanical platelets They also transport substances that assist in the coagulation process	[56]
6	Chromallocyte	The renovation machine will first assess the condition by inspecting the cellular substances, actions, and works These repair machines are capable of overhauling the complete cell	[57]

### Materials of nanorobots

At nanometer scales to work within tumor tissues and cells, the primary consideration for the design of nanorobots was the biocompatibilities of materials. The first challenge encountered in designing a nanorobot to perform medical tasks is the issue of materials science or surface science, since the operation of a microrobot is largely dependent on the properties of its surface and materials. The molecular interactions among biological species and the surfaces of a nanorobot drastically affects the motion control of a nanorobot in a biological microenvironment. Nanorobots are mostly made of biocompatible or biodegradable materials. These biodegradable materials are able to dissolve or disappear at the end of their tasks. Meanwhile, they should be able to accomplish a wide range of accurate tasks including sensing of the presence of tumor cells/tissues, delivery and release of nanocargoes upon stimulations upon physical cues, certain disease biomarkers, changes of local temperatures/pH values, etc. [58-61]. These materials should also be flexible and deformable to ensure workability and mechanical properties of nanorobots to work in the human biological microenvironments [62, 63]. They need to be more maneuverable in three dimensions, in viscous and elastic body fluids, as well as in phantom organs. Besides, when designing nanorobots to perform adaptive tasks in a variety of different biological environments, stimulating-responsive materials becomes significant important [64].

### Propulsion of nanorobots

The energy source of driving forces is vitally important for nanorobots to work in the body autonomously. The type of driving force can affect the moving speed of a nanorobot, controllability and biocompatibility to a great extent and thus subsequent applications in a biosystem. It is not possible to apply the conventional macroscopic batteries and power supply components to these nanorobots. In the design phase, it should be ensured that a nanorobot could move freely and has sufficient power to offset the resistance from TME (tumor microenvironment). The power sources of nanorobots are innovatively divided into exogenous dynamics and endogenous dynamics. Exogenous dynamics usually include magnetic propulsion, ultrasound propulsion, and light-driven propulsion, whereas endogenous dynamics are usually achieved by chemical or biological reactions [65-71]. Locomotional control also represents an important challenge in micro- and nanoscales. In vivo operations of nanorobots have been demonstrated their abilities to enhance tissue penetration and payload retention. But viscous forces dominate over inertial forces at nanoscopic scales. Therefore, it is necessary to take into account the environment effects while designing an efficient nano-machine. For example, it requires different swimming strategies that allow nanorobots to operate under these low Reynolds number constraints, as well as various kinds of navigation strategies for nanorobots to overcome the Brownian motion [9, 72-74].

Recently, blood glucose, urea and other bodily fluid constituents were utilized as the power sources for enzyme reaction-derived nanorobots, but the stability of these enzyme reactions-driven nanorobots requires further improvements before practical implementation can be possible [75, 76]. However, new alternative fuels and propulsion mechanisms are needed to achieve safe and successful operation in the human body, although different fuels and external stimuli have been explored for nanorobots in aqueous media [21, 77-79].

### Nanorobots core

After satisfying the previous fundamental requirements, an ideal nanorobot core is required. Much more research is needed before nanorobots can achieve widespread biological applications [80]. DNA origami is one of the greatest advancements in the core project of nanorobotics. A single-stranded DNA can be collapsed into a two-dimensional shape and eventually form a three-dimensional nanostructure, which can release its payloads upon binding with a specific cancer biomarker [81-85]. Viral capsids are robust and environmentally stable nanoparticles and are an innovative design employed by natural systems. The proteinaceous shell of viral capsids allows the protection of viral nucleic acids from denaturation by the external environment. The receptor, in turn, can be integrated into the face of the virus shell to allow initiation of conformational shifts in the shell structure and release of the nucleic acid into a selected host cell upon conjugation to the receptor’s corresponding biomarker or molecule [15, 86-88]. Another commonly adopted bioinspired technique for nanoparticle fabrication is chemical modification of natural polymers. Natural polysaccharides, such as chitosan, will be the most logical choice when we put biocompatibility at the top of the list. Chitosan has been widely used for nanoparticle productions in the last decade [75, 89-91]. Besides nanoparticles derived from chitosan, various kinds of nanoparticles, such as gelatin, alginate, pectin, chondroitin, and dextran, have been widely used in cancer therapies.

### Fabrication of nanorobots

When researchers design and build small-scale robots, they are motivated by the need to find active materials that can consistently convert different forms of energy into motion. The first generation of nano-engines for small-scale robots relied on their simple geometry and manufacturing procedures [92]. Through electrochemical reduction of salts corresponding to metals within nano/micron symmetric pores, these early nanorobots were fabricated [93]. Another strategy is the self-assembly of nanocomponents, for example, the layer-by-layer assembly of sequentially charged materials, the generation of self-organizing polymers to create bowl-shaped stomatal cells that can be filled with catalytic materials in their internal spaces, the attachment of colloids to create engineered structures and magnetic links [94-98].

Another strategy for fabrication of nanorobots is to use of a thin film layer on a template to produce an asymmetrical coating structure [99, 100]. From polymers to metal beads, these approaches make use of diverse commercially available microtemplates as well as biological and bioinspired templates [101]. 3D printing, glancing angle deposition, rolled-up lithography and other advanced techniques are also used in the design and fabrication of more complex nanorobots [102-106]. Each of these new innovations provides novel capabilities for design and high quality, although they are generally more expensive and have restricted material options.

Biohybrid nanorobots were fabricated with diverse methods. For example, in recent publications noncovalent interactions were commonly adopted to attach synthetic components to the head or tail of microorganisms [107]. Biohybrid nanorobots are made of living organisms and synthetic components, which were coupled together via electrostatic interactions-driven self-assembly. Another approach benefits from the physical retention of functional nanostructures on the rough surface of microorganisms [11]. But, due to the lack of covalent bonds between the synthetic material and the microbial surface, it tends to come off under certain environmental stresses.

### Degradation of nanorobots

Nanorobots may be manufactured and driven in a variety of ways, but toxin-free degradation is of great significance for biosafety. The degradability of materials in nanorobots is a key factor to be considered first before their biomedical applications [108-111]. High degradability could avoid the post-use operation of the nanorobots. For example, biodegradable polymers were adopted to make microrobots using laser direct writing to control the shape [112]. Water-soluble polymers such as polyvinyl alcohol were employed to produce drug-carrying biodegradable nanorobots in mass production [113]. Natural polymers including gelatin and chitosan could be hybridized with magnetic nanoparticles to produce magnetic field-driven, biodegradable nanorobots that could reach the targeted defective locus under proper magnetic field guidance payloads continuously upon being gradually degraded in a biological system, while the released payloads/cells could move toward the diseased site to execute repairment functions [114].

## Types of driving forces for nanorobots in cancer drug delivery

As nanotechnology keeps moving forward, drug delivery has become one of the most widespread functions of nanorobots in cancer therapy. Nano-drug carriers have been developed with some evident features, including small sizes, large specific surface area/internal void volumes, and outstanding physicochemical properties. An ideal nanorobot, in general, possesses some special capabilities such as controlled navigation, tissue penetration, propulsion, cargo hauling and release.

In addition to passive mass transport limitations, most existing drug delivery nanocarriers rely on systemic circulation, and they also lack the self-driving force and navigation capabilities required for targeted delivery and tissue penetration. Several anti-tumor therapies using nanorobots have been reported to enable precise therapeutic drug delivery to targeted tumor areas [114-121].

Nanorobots could be used to treat cancers via i.v. injection into the blood stream or uptaken via orally administration, gathering at the focus to significantly improve the anti-cancer effect with little harm to healthy normal cells. To accurately deliver the therapeutic payload directly to the tumor area, numerous advanced technologies were introduced to help nanorobots to reach the diseased sites. Nanorobots reported in recent years were categorized to a few different types based on their propulsion methods and motion driving forces.

### External magnetic-driven nanorobots

**Fig. 2:**
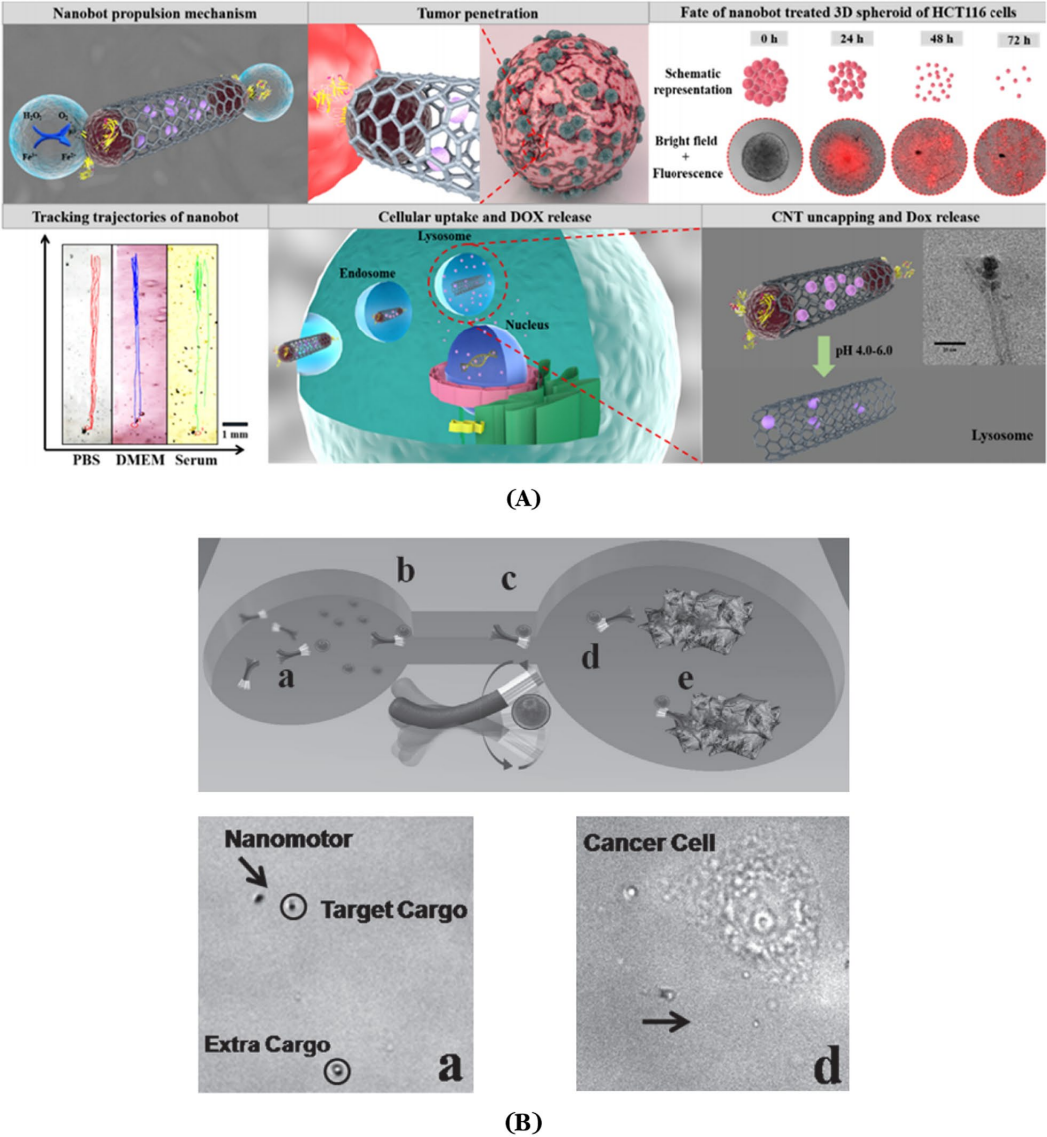
**A** Illustration of a DOX-loaded magnetic nanorobot which was driven to penetrate into the 3D spheroid tumor, followed by drug release under intracellular endo/lysosomal conditions. Modified and reprinted from ref. [131]. Reproduced with permission, Copyright 2020, Springer Nature. **B** The process for flexible magnetic nickel-silver nanoswimmer capturing magnetic polymer particles loaded with drugs and transporting it to target cells via channels. Modified and reprinted from ref. [132]. Reproduced with permission, Copyright 2011, Wiley–VCH

A number of preliminary studies have been performed to prove the transport function and properties of nanorobots using magnetic propulsion [122-126]. And research on the application of nanorobots for cancer treatment has also achieved numerous excellent outcomes. A prerequisite for this driving force pattern is that magnetic helically shaped nanorobots can be propelled by rotational-to-translational motion using a torque generated by an external magnetic field [127-130]. Andhari et al. [131] engineered a multi-component magnetic nanorobot, which was fabricated using multi-walled carbon nanotubes (CNT) loaded with doxorubicin (DOX) and anticancer antibody. This self-propelling magnetic nanorobot could be driven by an external magnetic field in complex biological fluids, and could release anticancer drug payloads inside the three-dimensional (3D) spheroidal tumors upon stimulation by intracellular H_2_O_2_ or local pH changes in the tumor microenvironments. The nanorobot was composed of chemically conjugating magnetic Fe_3_O_4_ nanoparticles and was able to preferably release DOX in the intracellular lysosomal compartment of human colorectal carcinoma (HCT116) cells via opening the gate on the surface of Fe_3_O_4_ nanoparticles (Fig. 2A). Wang et al. reported a nickel–silver nanoswimmer which could be powered by an external magnetic field and could deliver micron-sized particles at high speeds of more than 10 μm s^−1^ [132]. This modified polymer microspheres with doxorubicin were made of poly(D, l-lactic-co–glycolic acid) (PLGA). The robot was propelled by a flexible magnet and could deliver drug-carrying microspheres using its extended polydimethylsiloxane (PDMS) channel. When the nanoswimmer reached the vicinity of human cervical cancer (Hela) cells, the drug-carrying microspheres were released to kill the cancer cells (Fig. 2B).

Xie et al. adopted a biological template method and transformed pine pollens into a magnetic microrobot, filled doxorubicin into the natural cavity of the robot by vacuum loading and then used the collaborative behavior of the microrobot to upload drugs through the PDMS narrow channels. When reaching the interior space of cancer cells, the semi-natural magnetic microrobot uses its magnetic rotor inside the cavity to generate fluid and release the payload drug molecules to kill cancer cells [133]. Magnetic field-driven nanorobots are often used to imitate bacterial flagella motion with an external magnetic field and deliver anti-tumor drugs [134, 135]. Felfoul et al. found that biohybrid microrobots (based on magnetococcus marinus strain MC-1) can be successfully driven using an external magnetic field to deliver drug-loaded nanoliposomes to hypoxic regions within the tumor [136]. Bacteria in these natural environments are accustomed to swim along the magnetic field lines of the living region to areas with low oxygen content. When the drug-containing nanoliposomes are bound to MC-1 bacteria and administered into mice with xenogeneic neoplasms under the guidance of an external magnetic field, as much as 55% of the microrobots could penetrate the HCT116 large intestine anoxic area of tumors in a xenograft mouse model. As compared to passive reagents, the microrobot demonstrated an excellent xenograft tumor penetration ability.

### External ultrasound-driven nanorobots

**Fig. 3:**
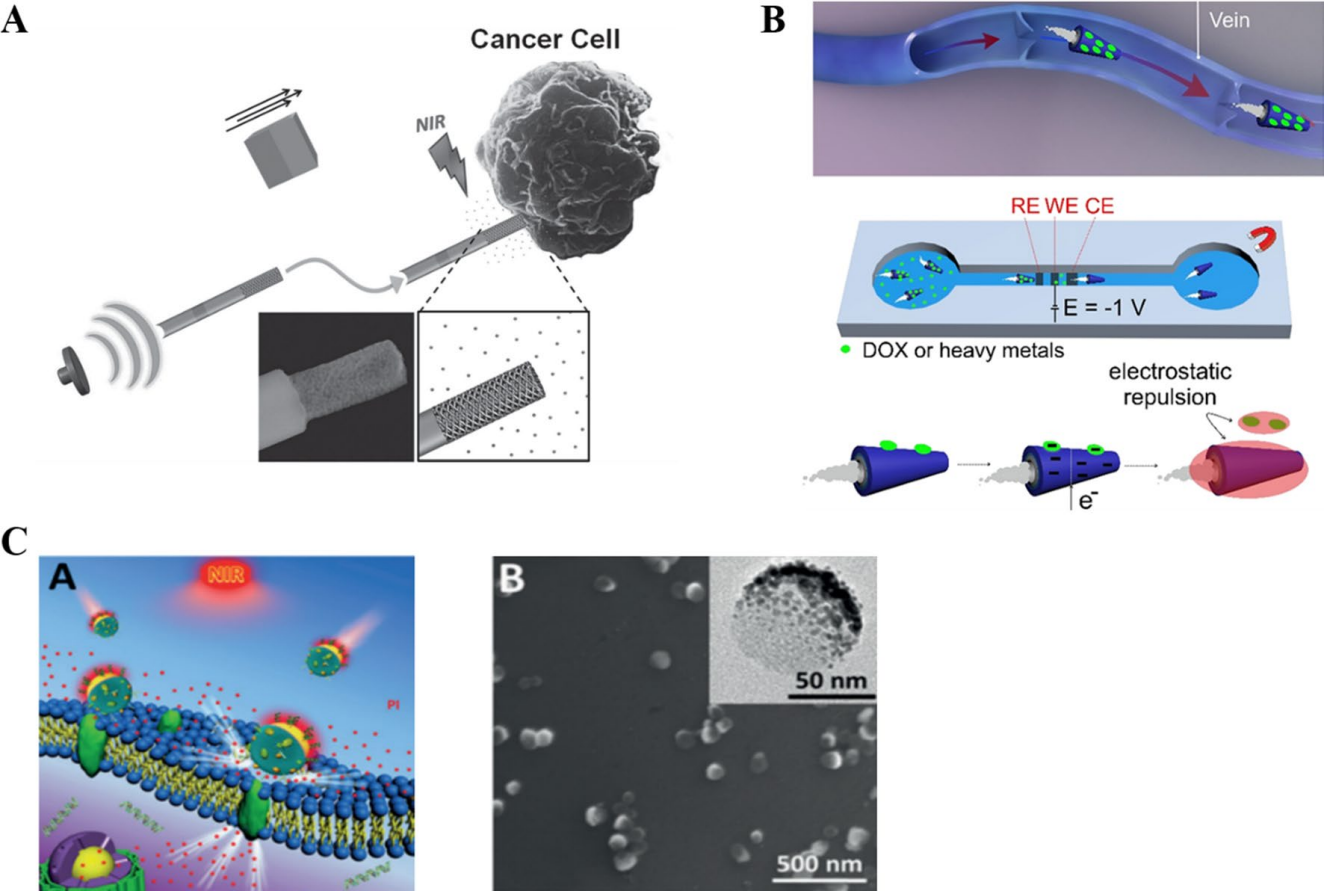
**A** Schematic of the nanowire motor which was driven by ultrasound toward cancer cells, followed by NIR light-triggered drug release. Modified and reprinted from ref. [139]. Reproduced with permission. Copyright 2014, Wiley–VCH. **B** The illustration of the Bi-based tubular microrobot showing the performance for smart drugs or heavy metals delivery in vein with electrochemical release mechanism. Modified and reprinted from ref. [147]. Reproduced with permission. Copyright 2019, American Chemical Society. **C** Schematic illustration of MPCM@JMSNMs applied to thermomechanically percolating the cell membranes, and the scanning electron microscopy (SEM) image of MPCM@JMSNMs. Modified and reprinted from ref. [148]. Reproduced with permission. Copyright 2018, Wiley–VCH

It is relatively easy to establish an acoustic condition. Being able to be transmitted in such media as solid, liquid and air, sound waves could penetrate deeply into biological tissues to power nanorobots from outside without causing noticeable harm to the human body. However, application of ultrasound may result in cellular oxidative stress, which may influence both target tumor cells and normal cells [137]. The underlying mechanism is that the ultrasonic wave exerts a local acoustic streaming strain on the surface of asymmetric nanorod-nanorobots, which generates a driving force for the movement of nanorobots. High-intensity focused ultrasound could be used to induce quick evaporation of chemical fuel and to drive tubular nanorobots in a flexible movement state. Such kind of microtube-based robots could move at an extremely high rate and penetrate into tissues with strong propelling force [138]. Garcia et al. [139] showed that ultrasound-driven nanowire motors could provide rapid drug delivery toward HeLa cancer cells to achieve a near-infrared light-triggered drug release. In this case, it was revealed that 38% of the DOX payload drug was released inside cancer cells after 15 min of NIR light irradiation (Fig. 3A).

### Biological-driven nanorobots

Bio-driven microrobots or nanorobots primarily refer to biohybrid microrobots. They are created from live microorganisms (cells) and artificial materials. Microorganisms, such as sperm and bacteria moving under the propelling effect of flagella, could act as an engine for biohybrid micro-/nanorobots. Besides, sperm, which has the special ability to bind with body cells, could significantly raise the issues of biocompatibility and safety of micro-/nanorobots. It was reported that a biohybrid robot having a 3D printed magnetic tubular microstructure and four arms adopts a mobile sperm cell as its power source and drug carrier. As compared to entirely synthetic microrobots or other nanocarriers, such kind of sperm-hybridized microrobots could seal high-concentration drugs into sperm membrane and protect the payload drugs from being diluted by body liquid or affected by enzymatic degradation [127].

### Hybrid-driven nanorobots

Many studies have testified the success of nanorobots in achieving targeted drug delivery using a hybrid power supply. Nanorobots were shown to have strong binding abilities toward pathogens and toxins, which allow them to achieve good detoxification abilities [140]. He et al. [141] built a tubular multi-layer microrobot using layer-by-layer self-assembly technology. With the combination of bubble drive and magnetic field guidance, these microrobots are able to rapidly deliver doxorubicin to cancer cells at speeds of up to 68 microns/second. The magnetic field could also control the motion of nanorobots along with other physical power methods [68]. For instance, Wang’s group from the University of California prepared porous metal rod-like nanorobots using an electrodeposition method [139]. The porous structure of their nanorobots makes it possible to carry more pharmaceutical molecules (20 times more than that by a planar metal counterpart). NIR (near-infrared) light was used to trigger the drug release from the nanorobots. Under the driving of ultrasonic wave and the guidance of an external magnetic field, tumor cells were effectively killed by the released drugs.

Chen et al. developed a hybrid magnetoelectric nanorobot able to execute targeted drug delivery in which drug release was triggered by an external magnetic field [142]. Victor et al. designed a magnetic field-guided three-segment Au–Ni–Au nanowire motor, which could be propelled by ultrasound [143]. The change of the applied magnetic field direction allows one to achieve omni-directional movement of the ultrasound-propelled particles. Bismuth (Bi) derivatives have been shown in recent studies to have promising abilities for biological applications [144, 145]. Beladi-Mousavi et al. presented the manufacturing of self-propelled Bi-based tube-like microrobots and demonstrated proof-of-concept experiments for intelligent drug delivery [146]. Bi was fabricated on the outer surface of the microrobots as drug carriers in clinical research. Bi-based microrobots were loaded with the clinical first-line anti-cancer drug DOX on their surface, and using the magnetic properties of the nickel layer, these robots were transported to target cancer cells. The loaded microrobots were directed by a magnet to a tunnel containing an electrochemical device, which allows the on-demand release of cargoes within only a few seconds [147] (Fig. 3B).

### Other power-driven nanorobots

Although it sounds promising, most reported examples were conducted in vitro levels. When dealing with a complicated inner biological environment, it remains to be proven whether the directional movement of nanorobots can be achieved as effectively as those shown in in vitro experiments. Xuan et al. [148] showed that a nanorobot system could perform a directional motion when exposed to NIR light (under NIR irradiation, Au half-nanoshells could produce a thermal gradient to provide self-heating energy to overcome Brownian motion). The NIR light-powered macrophage cell membrane-cloaked Janus mesoporous silica nanomotor (MPCM@JMSNMs) was also wrapped by macrophage membranes, giving the nanorobots the immunological property of selectively binding cancer cells (Fig. 3C). As micro-/nanorobots and their use in drug delivery have made great progress in recent research, these micro-/nanorobots are expected to be potent active delivery tools for various therapeutic applications that would otherwise be challenging to accomplish in current passive delivery systems.

## Medical nanorobots versus conventional nanomaterials for drug delivery

The emergence of nanotechnology has brought forth significant advancements in medical science, particularly in drug delivery. Conventional nanomaterials, also known as nanocarriers or nanomedicines, have been extensively used to improve drug delivery efficiency and specificity [149, 150]. Recently, a novel concept, medical nanorobots, has been introduced, showing great potential in cancer treatments and other biomedical applications [151]. This section will discuss the differences between medical nanorobots and conventional nanomaterials for drug delivery, with a focus on their design elements, targeting abilities, power systems, biocompatibility, safety considerations, and potential applications.

### Design elements

Medical nanorobots epitomize a groundbreaking innovation in the sphere of nanomedicine, functioning as nanoscale apparatuses specifically devised to execute an array of biomedical tasks, such as diagnostics, therapeutic interventions, and targeted drug delivery. Unlike conventional nanomaterials, which primarily serve as drug delivery vessels, nanorobots are engineered with a plethora of sophisticated components that empower them to operate efficaciously within the human body [151, 152]. Some of these components encompass a protective shell composed of biocompatible materials like silicon, carbon, or diamond, which safeguards the nanorobot and its internal components from the surrounding biological milieu. Moreover, nanorobots necessitate a power source, which can manifest as a battery, hydrogen fuel cell, or even energy harnessed from the body's metabolism, to fulfill their designated functions. The payload of a nanorobot alludes to its specific objective, such as targeted drug delivery, imaging, or tissue restoration [153, 154]. Furthermore, medical nanorobots are furnished with refined sensors that can detect alterations in the body, such as temperature, pH levels, or the presence of particular molecules, as well as actuators that permit them to physically interact with the body by traversing the bloodstream, dispensing drugs, or performing surgical procedures. Lastly, communication systems within nanorobots enable them to interface with each other or external apparatuses, such as computers or remote-control systems [20, 155]. In summation, medical nanorobots proffer a substantial advancement in nanotechnology, boasting an array of intricate design elements and functionalities that traditional nanomaterials do not have.

In contrast, conventional nanomaterials primarily function as vehicles for drug delivery, devoid of the intricate functionality and design constituents present in medical nanorobots. These nanomaterials are typically passive in nature and rely on the body's innate processes for their dispersion and release, which can constrain their effectiveness and specificity [156, 157]. Conventional nanomaterials may comprise liposomes, polymeric nanoparticles, or micelles, which encapsulate the therapeutic agents and safeguard them from degradation, but do not possess the advanced capabilities proffered by nanorobots, such as active propulsion, real-time sensing, or communication [158, 159]. This fundamental disparity in design and functionality between medical nanorobots and traditional nanomaterials underscores the potential of nanorobots to revolutionize drug delivery and other biomedical applications, proffering more targeted, efficient, and adaptable therapeutic resolutions.

### Enhanced targeting proficiencies

A salient advantage of medical nanorobots over conventional nanomaterials resides in their exceptional targeting capabilities, which can be largely ascribed to the intricate sensors incorporated into their design. These sensors endow nanorobots with the capacity to discern subtle fluctuations within the body, such as variations in temperature, pH levels, or the presence of specific biomolecules, encompassing proteins, enzymes, or other cellular markers [19]. Armed with this advanced sensory information, nanorobots can actively pursue and converge on target sites, including tumors, area of inflammation, or regions of infection, with remarkable precision.

In addition to sensors, medical nanorobots are also devised with advanced actuators and propulsion systems, permitting them to navigate and maneuver through complex biological environments, such as the bloodstream or interstitial spaces. This active propulsion enables nanorobots to surmount physiological barriers, access deeply-situated target sites, and permeate tissues more efficiently than their conventional counterparts [121, 160]. Consequently, therapeutic agents can be delivered with a high degree of precision, minimizing off-target effects and mitigating potential side effects. Furthermore, the amalgamation of these sophisticated design features empowers medical nanorobots to adapt and respond to the dynamic nature of the human body. They can modify their trajectory and delivery strategies in real time based on the sensed biological cues, ensuring optimal therapeutic outcomes. This adaptability and responsiveness are exclusive to medical nanorobots and proffer significant advantages over conventional nanomaterials, which are typically passive and contingent on the body's natural processes for distribution and release [161, 162]. In summary, the integration of intricate sensors, actuators, and propulsion systems in medical nanorobots enables more accurate and efficient delivery of therapeutic agents, resulting in improved treatment outcomes and diminished side effects.

In contrast, conventional nanomaterials primarily function as vehicles for drug delivery, devoid of the intricate functionality and design constituents present in medical nanorobots. These nanomaterials are typically passive in nature and depend on phenomena like the enhanced permeability and retention (EPR) effect, as well as the body's natural processes for their dispersion and release, which can constrain their effectiveness and specificity [163, 164]. Conventional nanomaterials may comprise liposomes, polymeric nanoparticles, or micelles, which encapsulate the therapeutic agents and safeguard them from degradation, but do not possess the advanced targeting capabilities proffered by the sensors in nanorobots.

This fundamental disparity in design and functionality between medical nanorobots and conventional nanomaterials underscores the potential of nanorobots to revolutionize drug delivery and other biomedical applications, proffering more targeted, efficient, and adaptable therapeutic resolutions. The superior targeting performance of medical nanorobots has the potential to transform drug delivery and various other biomedical applications, providing more precise and efficient therapeutic solutions with minimal off-target effects. This could lead to enhanced patient outcomes, reduced side effects, and the development of innovative treatment strategies for a wide array of diseases and conditions.

### Active versus passive power systems

The primary distinction between medical nanorobots and conventional nanomaterials resides in their respective power systems. Medical nanorobots are furnished with active power systems, empowering them to harness external power sources such as near-infrared light, ultrasound, or magnetic driving forces. Additionally, they can capitalize on the inherent flow of biological mediums, like blood, to traverse the body [165, 166]. This active propulsion capability considerably amplifies their maneuverability and navigation, permitting them to efficiently reach specific targets and execute their designated functions with remarkable precision.

Conversely, conventional nanomaterials lack active power systems, relying instead on passive mechanisms such as diffusion or convection to navigate through biological systems. These passive transport methods intrinsically circumscribe the mobility and functionality of nanocarriers, rendering them less efficient and versatile in certain drug delivery applications when juxtaposed with their nanorobot counterparts [167, 168]. The absence of an active power system in conventional nanomaterials may culminate in sluggish transport, diminished targeting accuracy, and reduced control over the release and distribution of therapeutic agents.

The active power systems inherent in medical nanorobots endow them with superior mobility and functionality as compared to conventional nanomaterials, which are contingent on passive transport methods. This fundamental distinction enables nanorobots to excel in various drug delivery applications, proffering enhanced targeting, precision, and control over the delivery and release of therapeutics. Consequently, medical nanorobots hold tremendous promise for revolutionizing drug delivery and other biomedical applications, potentially leading to improved treatment outcomes and reduced side effects for patients.

### Biocompatibility and safety considerations

A pivotal aspect distinguishing medical nanorobots from conventional nanomaterials is the imperative to ensure biocompatibility and safety. Owing to their elaborate design and intended interactions with intricate biological systems, nanorobots must be fabricated with biocompatible materials and components to circumvent eliciting adverse reactions or immune responses. This requirement necessitates meticulous contemplation of various factors, such as the selection of materials, surface chemistry, and potential for long-term accumulation within the body. Furthermore, addressing the potential risks of toxicity and clearance of nanorobots is crucial to protect patient health [169, 170].

While conventional nanomaterials are also mandated to be biocompatible, their relatively simpler design and fewer interactions with the body result in a less convoluted safety profile. Conventional nanomaterials generally comprise well-established materials such as liposomes or polymeric nanoparticles, which possess a more linear safety evaluation process [171, 172]. Nonetheless, both medical nanorobots and conventional nanomaterials must undergo rigorous safety assessments, preclinical testing, and regulatory approval before being deployed in clinical applications.

The biocompatibility and safety considerations for medical nanorobots underscore the challenges and opportunities in developing these advanced technologies for clinical use. As research advances and safety concerns are addressed, medical nanorobots harbor the potential to revolutionize drug delivery and other biomedical applications, providing more targeted, efficient, and adaptable therapeutic solutions for a diverse array of diseases and conditions.

### Potential applications and future outlook

While conventional nanomaterials have already been employed in numerous drug delivery applications, medical nanorobots offer a vast array of potential uses that remain under exploration. These applications encompass targeted drug delivery, wherein nanorobots can precisely deliver therapeutics to specific tissues, cells, or even subcellular locations, thereby minimizing side effects and maximizing treatment efficacy [173]. In vivo diagnostics is another potential application, with nanorobots being utilized for real-time monitoring of various physiological parameters, which can aid in the early detection and diagnosis of diseases [174]. Furthermore, nanorobots could revolutionize regenerative medicine by assisting in tissue repair and regeneration, as well as modulating the immune system by suppressing it in autoimmune diseases or enhancing its anti-tumor activity in cancer treatments. Medical nanorobots could also be harnessed for minimally invasive microsurgical procedures, allowing for more precise and targeted interventions, reduced trauma, and faster recovery times [11, 175]. As the field of medical nanorobots continues to advance, their potential applications are anticipated to expand even further, transforming the landscape of medicine and providing innovative therapeutic options for a wide range of diseases and conditions.

In summary, medical nanorobots and conventional nanomaterials differ significantly in their design elements, targeting abilities, power systems, biocompatibility, safety considerations, and potential applications. While conventional nanomaterials have been successfully utilized in drug delivery applications, the emergence of medical nanorobots holds immense promise for an array of novel biomedical applications, including cancer treatment, diagnostics, tissue repair, immune system modulation, and microsurgery [19, 120]. As research and development in this field progress, medical nanorobots may ultimately transform the landscape of medicine and provide new therapeutic strategies for various diseases, enhancing patient outcomes and overall healthcare quality.

## Nanorobot-assisted cancer diagnosis and targeted therapies

As compared to traditional anti-cancer treatments, emerging targeted therapy can selectively interact with specific biomarkers that are involved in tumor development, and obstructs tumor growth [176]. It provides certain significant advantages over traditional chemotherapy, as it targets only at those specific biomarkers related to tumor growth. In recent years, several anti-tumor therapies have been reported to achieve targeted therapy using nanorobots [173, 177-180]. Nanorobots-assisted targeted therapy could avoid unwanted side effects of high toxicity besetting traditional chemotherapy, and provide a new solution for anti-cancer treatment [181]. Nevertheless, power-driven nanorobots could target at specific lesions, implement controllable movement, detection, positioning and gathering, as well as administer therapeutic compounds in a proper and targeted manner [182, 183]. Some common directions of clinical applications of nanorobots in future cancer diagnosis and treatments are briefly summarized in Table 3. We also summarized the recent ongoing clinical trials about the applications of nanorobots or nanomedicine on cancer treatment (Table 4). Nanorobotics is an interdisciplinary field that combines the principles of robotics, nanotechnology, and material science to develop robots at the nanoscale. The use of nanorobots could lead to significant advancements in fields like medicine, manufacturing, energy production, and environmental cleanup. Nanorobotics could also lead to new scientific discoveries and a deeper understanding of the nanoscale world. However, after an extensive review of relevant literature and a comprehensive search for clinical trials related to nanorobots for cancer treatment on the website of www.clinicaltrials.gov (Additional file 2), we realized that much of our current knowledge about nanorobotics is theoretical and conceptual, or still in the preclinical research stage. As such, there are still very few absolute nanorobots with all 5 components mentioned in Table 1 currently in clinical uses.

**Table 3:** Common directions of clinical applications of nanorobots in future cancer diagnosis and therapeutic treatments

Entry	Application	Explanation	Advantages	Limitations
1	Targeted imaging	Nanorobots can be engineered to selectively target at cancer cells/tumor tissues, allowing for improved imaging and visualization of the tumor	Enhanced imaging and visualization of a tumor, providing more accurate diagnosis Increased specificity in targeting at cancer cells, reducing harm to healthy cells Potential to visualize smaller tumors or lesions that may be missed by traditional imaging techniques Improved ability to monitor treatment response and track changes of a tumor over time	Technological difficulties in engineering nanorobots to effectively and selectively target at cancer cells or tumor tissues The cost and intricacy of producing and deploying substantial numbers of nanorobots
2	Tumor biopsy	Nanorobots can be designed to perform minimally invasive biopsy procedures, allowing for the collection of tissue samples for diagnosis	Minimally invasive biopsy procedures, reducing the risk of complications and patient discomfort Improved accuracy in collecting tissue samples, providing a more precise diagnosis Potential to reach and collect bio-sample previously inaccessible tumors	The challenges faced in creating nanorobots that can successfully carry out biopsy procedures due to technical limitations The expenses and complexities involved in manufacturing and utilizing large quantities of nanorobots
3	Molecular diagnosis	Nanorobots can be engineered to perform molecular diagnostics, allowing for the early detection of specific cancer biomarkers and improved diagnosis	Improved accuracy in detecting specific cancer biomarkers, providing a more precise diagnosis Increased efficiency in performing molecular diagnostics, reducing the time and cost of diagnosis Potential to detect cancer at an earlier stage, improving patients’ prognosis outcomes	Challenges posed by technology in designing nanorobots for molecular diagnostic purposes Financial and technical hurdles involved in manufacturing and distributing significant amounts of nanorobots
4	Targeted administration	Nanorobots can be engineered to selectively target at cancer cells or tumor tissues, thereby enhancing the efficacy of immunotherapy and minimizing adverse effects	Improved efficiency in directing immunotherapy agents directly to the tumor site Lessened adverse effects as compared to systemic drug administration methods Administering higher doses of immunotherapy agents to the tumor site, thereby improving treatment efficacy Enhanced specificity in targeting at cancer cells/tumor tissues, thus reducing harm to healthy normal cells	The intricacies involved in engineering nanorobots with the capability to effectively and precisely target at cancer cells/ tumor tissues The expenses and complexity associated with the mass production and deployment of these nanorobots
5	Continual monitoring	Nanorobots can be designed to continuously monitor the local changes of a tumor and release immunotherapy agents as required, thus providing a more adaptive and dynamic therapeutic treatments	Providing a more adaptable and dynamic therapeutic treatment method, responding to changes in the tumor microenvironment Capacity to continuously monitor the tumor and release immunotherapy agents as necessary, thus enhancing treatment efficacy	Technological difficulties in designing nanorobots with the capability to continuously monitor a tumor, and release immunotherapy agents The cost and intricacy of producing and deploying substantial quantity of nanorobots
6	Conjoint therapy	Nanorobots can be engineered to administer multiple immunotherapy agents simultaneously, thereby allowing for a more comprehensive and effective cancer treatments	Potential to simultaneously deliver multiple immunotherapy agents, thereby enhancing treatment efficacy Ability to exert targeted delivery of immunotherapy agents, reducing harm to healthy normal cells Improving patient outcomes through the integration of multiple treatments into a single nanorobotic platform	The technical challenges in designing nanorobots capable of delivering multiple immunotherapy agents with high efficacy The financial implications and intricacies involved in the large-scale production and deployment of nanorobots
7	Tumor ablation	Nanorobots can be designed to physically destroy cancer cells through various means such as heat, light or mechanical ablation	Potential to directly and physically destroy cancer cells, reducing the risk of tumor recurrence Ability to destroy cancer cells in areas that are difficult to access using traditional surgical tools or methods Potential for minimally invasive treatment with reduced risk of complications as compared to traditional surgery	Technical challenges in designing nanorobots to accurately destroy cancer cells Cost and complexity of manufacturing and deploying large quantity of nanorobots

**Table 4:** Some important clinical trials about the applications of nanorobots or nanomedicine on cancer treatments

Study	NCT number	Title	Status	Clinical trial outcome	Conditions	Interventions	Outcome measures	Gender	Age	Phases	Enrollment	Study type	Study designs	Start date	Primary completion date	Completion date	Country
1	NCT04918381	CellFX Treat & Resect Low-Risk BCC Feasibility Study	Completed	The study included 30 participants with a total of 37 BCC lesions. The primary purpose of the study was treatment, and there was no control group. This study found that the CellFX system was safe and feasible for the treatment of low-risk BCC lesions, with no serious adverse events reported. The trial was sponsored by Pulse Biosciences, Inc. and was completed in 2022	BCC—Basal cell carcinoma BCC excision margin	Device: CellFX system	Number of lesions with BCC histological clearance; number of participants with treatment related serious adverse events	All	22–85 years (Adult, older adult)	Not applicable	30	Interventional	Allocation: N/A; intervention model: single group assignment; masking: none (open label); primary purpose: treatment;	02-Jun-21	02-Mar-22	22-Jul-22	United States
2	NCT04789486	Nano-SMART: nanoparticles with MR guided SBRT in centrally located lung tumors and pancreatic cancer	Recruiting	No final result was reported yet	Non-small cell lung cancer, advanced pancreatic adenocarcinoma, unresectable pancreatic cancer, ductal adenocarcinoma of the pancreas	Drug: AGuIX, radiation: radiotherapy	Maximum tolerated dose (MTD), Phase 1, compare local control at month 12^th^ of maximum tolerated dose MTD—Phase 2, progression-free survival (PFS) at maximum tolerated dose (MTD), overall response rate (ORR) at maximum tolerated dose (MTD), serious adverse events at day 90^th^, serious adverse events at month 12^th^, tumor changes, compare disease-specific survival, compare R0 resection rate, compare overall survival, quality of life (QoL)-performance status utilizing PROMIS physical and mental health batteries, quality of life (QoL)-completion of daily activities utilizing PROMIS physical and mental health batteries	All	18 years old and older (adult, older adult)	Phase 1, Phase 2	100	Interventional	allocation: randomized, intervention model: parallel assignment, masking: none (open label), primary purpose: treatment	27-May-21	10-Apr-23	10-Sep-24	United States
3	NCT05340725	Rectal dexmedetomidine niosomes for postoperative analgesia in pediatric cancer patients	Recruiting	No final result was reported yet	Postoperative pain	Drug: DEX-IV; drug: DEX-Rectal; drug: DEX-Nano-Rectal	Serum concentrations of Dexmedetomidine, postoperative FLACC pain score	All	3–7 years old (children)	Phase 2, Phase 3	45	Interventional	Allocation: randomized, intervention model: parallel assignment, masking: quadruple (participant, care provider, investigator, outcomes assessor, primary purpose: treatment	1-May-22	1-Dec-23	1-Dec-23	Egypt
4	NCT04759820	Carbon nanoparticles vs indocyanine green	Recruiting	No final result was reported yet	Number of lymph node retrieved	Drug: carbon nanoparticles suspension, drug: indocyanine green	Number of lymph nodes detected; number of positive lymph nodes detected at different T stages, the ratio of positive lymph nodes, patients’ disease-free survival (DFS)	All	18–70 years old (adult, older adult)	Phase 2, Phase 3	298	Interventional	Allocation: randomized, intervention model: parallel assignment, masking: none (open label), primary purpose: prevention	1-Jan-21	30-Jun-22	30-Jun-23	China
5	NCT04881032	AGuIX nanoparticles with radiotherapy plus concomitant temozolomide in the treatment of newly diagnosed glioblastoma	Recruiting	No final result was reported yet		Drug: polysiloxane Gd-chelates-based nanoparticles (AGuIX), radiation: radiotherapy, drug: Temozolomide	The recommended dose (phase I) of AGuIX in combination with TMZ and radiotherapy during the radio-chemotherapy period, 6-month progression-free survival (PFS) rate (phase II), pharmacokinetic C_max_ of AGuIX, pharmacokinetic T_max_ of AGuIX, pharmacokinetic AUC of AGuIX, pharmacokinetic t_1/2_ of AGuIX, distribution of AGuIX, overall survival, progression-free survival (PFS), toxicity (CTCAE criteria)	All	18–75 years old (adult, older adult)	Phase 1, Phase 2	66	Interventional	Allocation: randomized, intervention model: parallel assignment, masking: none (open label), primary purpose: treatment	7-Mar-22	Sep-24	Mar-26	France

### Cancer detection and diagnosis

Early detection of cancers is urgently needed because it can increase greatly the survival rates for patients [184]. The study on tumor-killing nanorobots keeps moving forward, accompanied by increasingly mature designs of nanorobots, leading to more effective and accurate early-stage clinical cancer diagnosis [185-188]. Maheswari et al. [189] proposed another tumor-detecting nanorobot that could examine tumor cell growth in vivo using positron emission topography. In the meanwhile, an embedded system was embedded so that the nanorobot could be controlled through pre-programmed procedures on the Arduino software platform. In order to avoid any potential side effects on the human body, an isotope-labeled nano-carbon material was used to fabricate the nanorobot. After being injected into a human body, nanorobots will not cause any harm to the human body with their reliable stability and safety. Once accomplishing the pre-set tasks, the nanorobot will be discharged from the human body as excrement. Similar to macro-robot in composition, the nanorobot is also composed of sensor, power device, and a camera. In addition, advanced algorithms were employed to design the shortest path, and the build-in sensor helps the nanorobot to evade from obstacles.

Overexpressed biomarkers on the cancer cell membrane surface provide good targets for disease diagnosis, therapeutics and biomedical engineering [190-192]. Peng et al. designed and engineered an 3D DNA nanorobot [193]. This 3D DNA-based logic gate nanomachine was designed to target at overexpressed cancer cell biomarkers with bispecific recognition [194, 195]. Besides, the DNA nanorobot can perform Boolean logic operations on the cancer cell membrane and has a great theranostic potential to be used in clinical treatment of cancers. Dolev et al. [196] designed a nanorobot that could detect circulating cancer cells in the blood and expose the drug to the tumor site under the driving force. This nanorobot was capable of storing electricity in a built-in capacitor, and it had the ability to harvest blood energy. The glucose levels in cancer cells are usually higher than those in normal cells. A high glucose level can promote cancer cells proliferation and metastasis [197]. A glucose sensor was immobilized on a CNT-based nanorobot to detect cancer cells via the elevated level of glucose-driven electric current in cancer cells. At the same time, this mechanism could in turn permit the activation of a nanoelectromechanical (NEM) relay (mechanical transistor) by the electric current, and it could break the chamber ceiling, exposing a drug identified by the immune system for cell elimination. This concept is in line with the effort on designing an autonomous computational nanorobot for in vivo medical diagnosis and treatment.

**Fig. 4:**
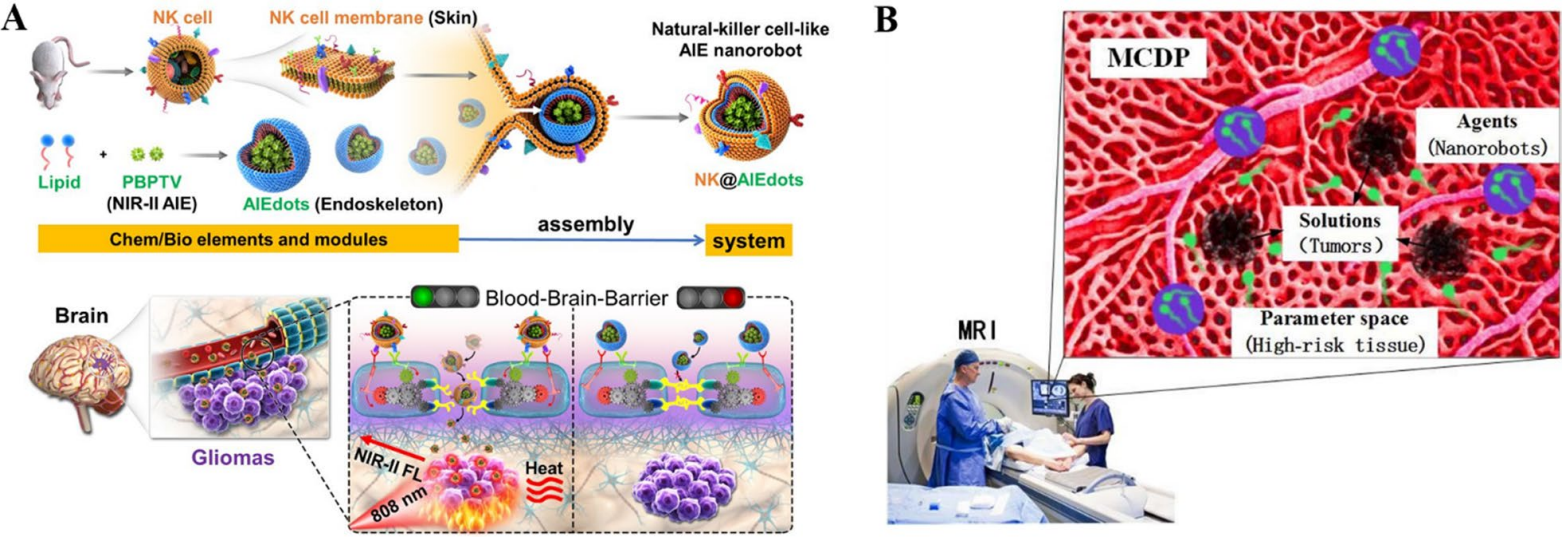
**A** Diagram showing the preparation and assembly processes of (NK@AIEdots) and the “smart tight-junctions (TJs)-modulated BBB penetration of NK@AIEdots for brain tumor-targeted light-up and inhibition. Modified and reprinted from ref. [205]. Reproduced with permission. Copyright 2020, American Chemical Society. **B** Pictorial illustration of the MCDP model. Modified and reprinted from ref. [206]. Reproduced with permission. Copyright 2020, IEEE

As part of the innate immune system, natural killer (NK) cells are lymphocytes and can breach the BBB (brain blood barrier) by using certain membrane proteins [198-202]. NK cells were used for cancer immunotherapy as previously reported [203, 204]. Deng et al. developed NK cell-mimic nanorobots with aggregation-induced emission (AIE) character (NK@AIEdots) by wrapping NK cell membrane on an AIE-active polymeric nanoendoskeleton [205]. The nanorobots have good biocompatibility and could emit very strong fluorescence in the NIR-II region upon photo-excitation. Besides, they could move across the brain–blood barriers in a self-help manner by unzipping tight junction structures and specifically accumulate at brain tumor sites in the complex brain matrix to provide tumor imaging with high contrast and penetration into the skull (Fig. 4A).

Shi et al. proposed a nanorobot-assisted multifocal cancer detection procedure (MCDP) which adopted a niche genetic algorithm (NGA) technology for multifocal cancer detection [206]. The NGA-inspired nanorobots, which detect tumors by swimming in the high-risk tissue region, can be regarded as an auto-searching process where the system could search for the optimal solution of an objective function in the parameter space with some constraints. It can solve the multimodal optimization (MMO) problem to locate at the tumor sites efficiently while considering realistic in vivo propagation and controlling of the nanorobots (Fig. 4B).

Wang et al. [207] reported a DNA logic-gated nanorobot (DLGN) that can anchor on the surface of living cell membranes to load multiple inducers and therapeutic agents for effective and precise treatment approaches. This nanorobot not only facilitated precise detection among five cell lines, but also exerted effective killing of cancer cells via triggered release of effector aptamer-tethered synergistic drugs (EASDs) in the cancer cells. The logic-gated recognition integrated into inducer-functionalized molecular machines enables rapid cancer dissection, in situ trapping and separation, and the safe delivery of precision medicine.

With a diameter of less than 100 nm, nanoparticles can move across various biological barriers, such as the brain–blood barrier or gastrointestinal barrier, which is an unique feature of nanorobots for detecting and diagnosing tumor cells at a very early stage, ideally at the level of a single cell or multiple cell level [208, 209]. These tumor searching-&-detection nanorobots exhibited excellent tumor-targeting efficiency for precise localization of cancer cells.

### Targeted delivery of nucleic acid for gene therapy

**Fig. 5:**
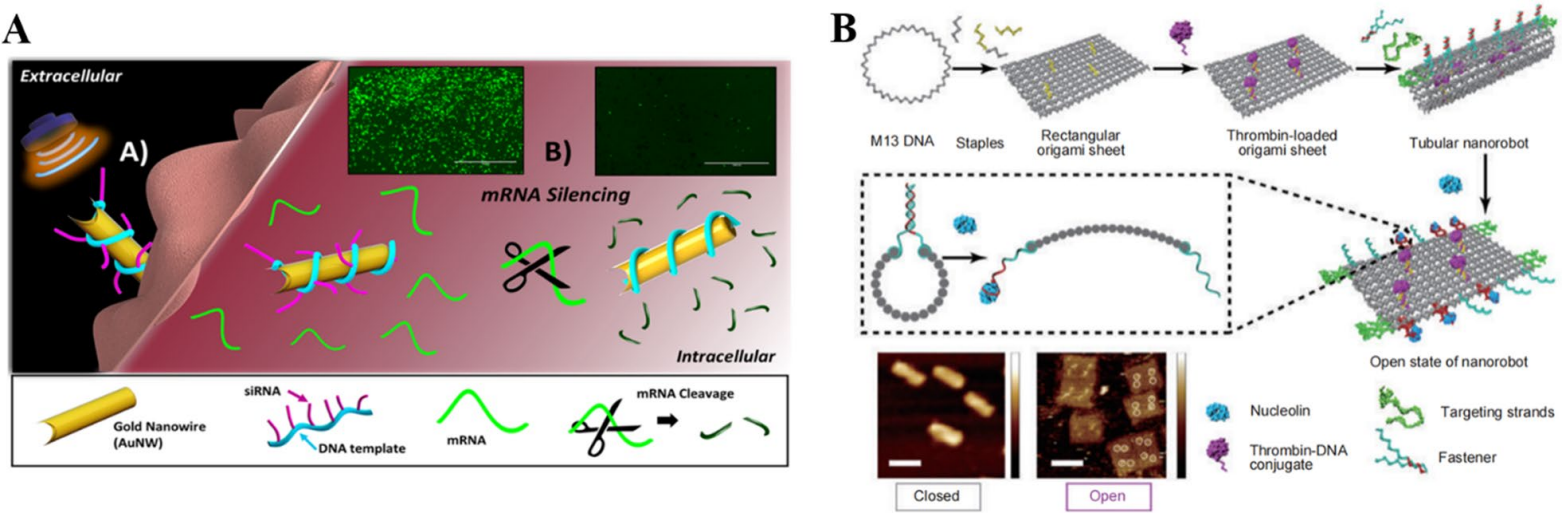
**A** Schematic illustration of the steps of the gold nanowire driven into living cells by an extracellular ultrasound field. And the images of gene-mRNA silencing in living cells. Modified and reprinted from ref. [210]. Reproduced with permission. Copyright 2016, American Chemical Society. **B** Illustration of the process of the thrombin-loaded DNA origami nanorobot bending into specific tubular nanorobot, opening and releasing the thrombin after the nanorobot reaching the nucleolin-binding aptamer. Modified and reprinted from ref. [212]. Reproduced with permission, Copyright 2018, Springer Nature

Except for drugs, targeted delivery of various theranostic compounds using nanorobots could avoid unwanted side effects besetting conventional chemotherapy such as high toxicities, and provides a new solution for anti-cancer treatments. For example, magnetic helical microswimmers can be directed delivery of pDNA to fetal kidney cells of human. Motors loaded with pDNA are wirelessly guided to the cell to release their gene cargo into the cell upon exposure. A gold nanowire coated with a rolled amplified DNA strand that can hybridize with siRNA was engineered for delivery of intracellular siRNA [210]. The pressure gradient generated by ultrasound provides a fast and strong thrust for the motion of the nanorobot, thus allowing the nanorobot to effectively penetrate into the cancer cells, and then, the target mRNA is split by the scissor-like scissors of siRNA, which is able to reach 94% efficiency of silencing in a few minutes of processing (Fig. 5A).

In 2017, Thubagere et al. [211] reported a DNA nanorobot equipped with a sorting function. The DNA nanorobot was designed with two walking legs and cargo-carrying arms. It could pick up the target cargo during the trip and deliver the cargo to target sites. As illustrated by experiments, this nanorobot could deliver cargoes to a targeted site with a success rate of 80%. It means when applied to tumor treatment, it could deliver drugs in a human body and kill the tumor cells at 80% success rate. However, in clinical applications, there is still 20% uncertainty, suggesting that some unknown harm may be brought to the healthy normal cells of a human body. Once receiving the command from an external control system, the nanorobot could unload the drug; if otherwise, it would keep moving without releasing the payload drug. DNA nanorobots could communicate with each other depending on algorithms, find the hiding places of tumor cells, and kill them with their payload drug. The design of this tumor-killing nanorobot relies more on biological molecules existing in a human body, so it can adapt to a complicated environment there.

### Infarction of tumor blood vessels

DNA-guided thrombin-inducing nanorobot is becoming a powerful treatment strategy for cancer [84, 212-214]. The technology has shown promising anticancer efficacy with low toxicity in preclinical settings. Translational studies of this technology in clinical trials represent a major advance in the application of DNA nanotechnology for anticancer therapy. In 2018, Li et al. [212] designed a kind of DNA origami nanorobot using the DNA origami technique. Depending on the DNA origami technology, Li et al. also suggested a customized tubular DNA nanorobot that could be bent into a specific conformation [212]. Within the tube, thrombin was loaded and isolated from the external circumstance so that it would not be enzymatically degraded during the transportation. Besides, a smart system was built into the DNA nanorobot so that it became biologically specific and could find the hiding places of tumor cells with high precision. DNA nanorobot found its right target through the specific receptor on the tumor cell membrane surface. After reaching the receptor, a build-in molecular switch was activated to release the thrombin within the right vessel to block the blood vessel and forbid nutrient supply to tumor tissues (Fig. 5B).

As an acute blood event, DNA-guided thrombin-inducing nanorobot was demonstrated to induce quick and massive necrosis of tumor cells with a more dramatic efficacy than many other therapies. Besides, it has no fatal side effects to the heart and does not cause any detectable damages on vital organs as compared to other effective anticancer modalities. (such as life-threatening cardiovascular toxicities from chimeric antigen receptor T (CAR-T) cell immunotherapy) [215-217]. However, several potential clinical concerns were raised for the DNA-guided thrombin-inducing nanorobot recently [218]. Vasculogenic “rebounds” may appear after tumor vascular infarction caused by the DNA nanorobot, in addition to an increased risk of tumor lysis syndrome (TLS) which results in serious metabolic crisis [219-224].

### Other targeted cancer therapies

#### Various DNA nanorobots

**Fig. 6:**
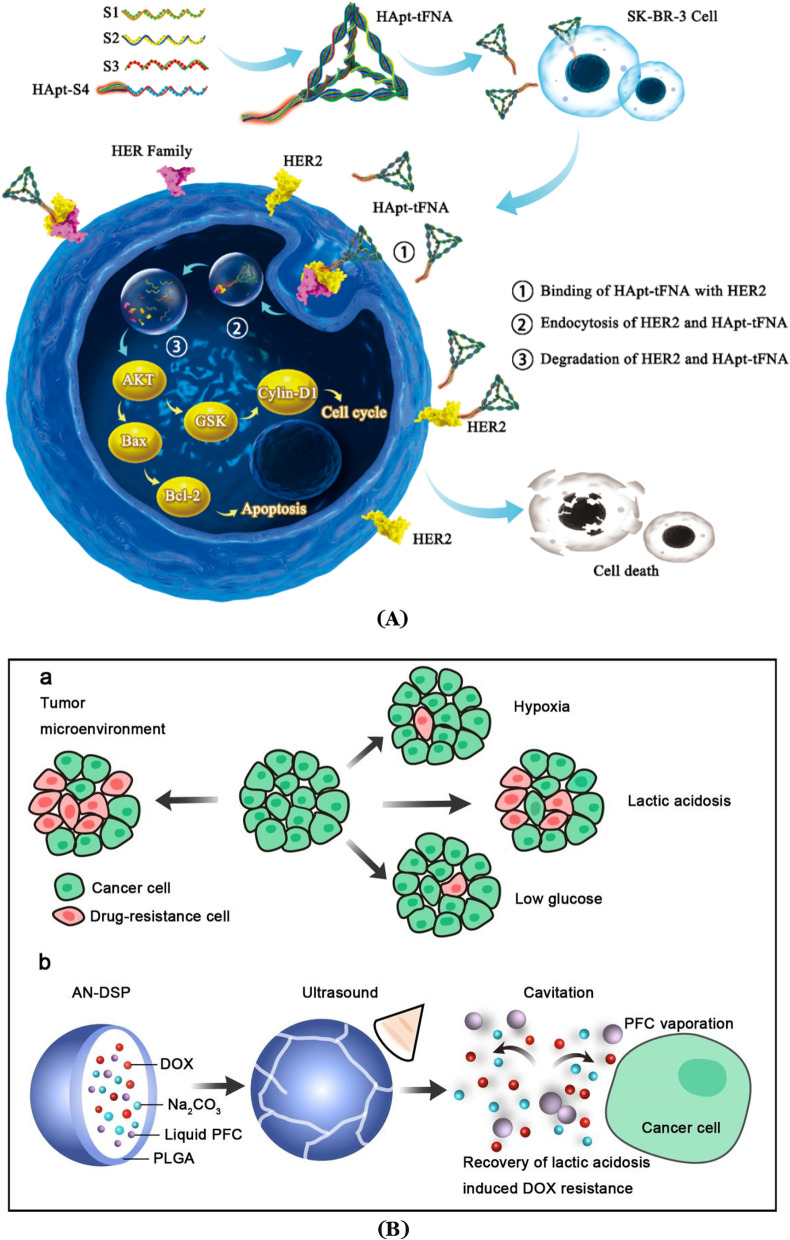
**A** Schematic illustration of the nanorobot HApt-tFNA incorporating HER2 in the HER2-HApt-tFNA complex and internalizing the complex into human mammary gland adenocarcinoma cells. The complex degrades within lysosomes, which suppresses cell proliferation and induces cell death. Modified and reprinted from ref. [249]. Reproduced with permission. Copyright 2019, American Chemical Society. **B** The effects of the cancer microenvironment on the development of drug resistance, and the ultrasound-responsive alkaline nanorobots (AN-DSP) for enhancing anticancer effects. Modified and reprinted from ref. [268]. Reproduced with permission. Copyright 2020, Royal Society of Chemistry

DNA-based nanorobots are inherently biocompatible and biodegradable, and they have attracted great attention owing to their high potential in various applications for cancer treatment [225-230]. Based on the classic principle of complementary base pairing, a single-stranded DNA is folded repeatedly and fixed by many short “staple strands” oligonucleotides to obtain the designed DNA nanostructures. DNA origami has excellent addressability to allow additional functional ligands, biomolecules, or nanoscale objects be organized precisely on a desired position along its outer surface, which introduces the targeting ability to DNA origami nanorobots [231-239]. Li et al. designed a pre-programed rectangular DNA origami nanorobot (20 nm × 30 nm), which could load with Adriamycin and effectively penetrate into ovarian cancer cells [240]. DNA origami nanorobots have also been reported to deliver ribonuclease (RNase) A molecules successfully into cancer cells [212, 241]. Singh et al. argued that DNA single-strand could be bent into a proper shape with DNA origami technology [242]. Some chemical approaches could be adopted to change DNA’s molecular properties so that the tubular nanorobot could accurately bind with the receptor in vivo and achieve site-selective treatment purpose. However, it is also believed if blood supply within tumor cells remains insufficient, the DNA nanorobot may not be able to achieve a significant effect on the treatment. HER2, as one of the transmembrane epidermal growth factor receptors (EGFRs), is involved in various types of information signaling processes among cancer cells. HER2 can enhance the malignancy of breast cancer. Overexpression of the HER2 receptor always leads to a poor prognosis for affected individuals [243-246]. In order to improve its delivery and therapeutic efficacy in treating HER2-positive breast cancer with less side effects [247, 248], Ma et al. [249] designed a nanorobot called HApt-tFNA, in which he anchored an anti-HER2 inducer (HApt) to a tetrahedral framework nucleic acid (tFNA). The nanorobot’s composition is based on DNA framework smart DNA, which can selectively degrade specific tumor proteins in cancer cells. The DNA nanorobot was then injected into a mouse model. Experimental results showed that the presence of tFNA could enhance the stability of the DNA nanorobot and prolong the blood circulation time of HApt. The HApt-tFNA could therefore drive HER2 into lysosomal degradation with higher efficiency (Fig. 6A). This novel DNA nanorobot opens up a new path for targeted protein degradation in precision breast cancer treatment, and improves the prognosis of breast cancer patients.

#### Photothermal therapy

As a photothermal therapy with high spatiotemporal selectivity, NIR light can be absorbed by nanorobots and converted to local thermal heat to induce cancer cell apoptosis process [250-252]. However, lack of precise and controllable targeting to tumor sites hinders the development of photothermal therapy [253, 254]. Song et al. developed a NIR light-responsive drug-loaded robust magnetic tri-bead microrobots and demonstrated their good biocompatibility even when their concentration is up to 200 µg/mL [255]. The microrobots showed fast NIR-responsive photothermal property in in vitro experiments. Microrobots were triggered to release payload drugs when the local temperature reaches 50 °C. The microrobots inside the microchannel could target tumor cells, and the successful application of targeted chemotherapy-photothermal therapy to lung cancer cells in vitro demonstrated the feasibility of nanorobotic targeted chemotherapy-photothermal therapy in cancer treatment.

#### Nanorobot-assisted detoxification

Nanorobots are also used as a dominant detoxification tool with excellent cleaning capabilities. As with biosensing, the detoxification approach relies on self-propelled nanorobots that rapidly arrest and eliminate toxins to reduce environmental nontoxicity. Esteban-Fernández et al. [140] reported a nanorobot for multipurpose removal of biological threat agents, particularly for biodetoxification and concurrent removal of pathogenic bacteria and toxins. This dual–cell membrane–functionalized nanorobot was integrated with diverse biological functions from the plasma membranes of two cell types, namely red blood cells (RBCs) and platelets (PLs).

Drug resistance is one of the major challenges in the treatment of malignant tumors [256-258]. In the tumor microenvironment, lactic acidosis plays a critical role in the development of drug resistance, which makes it an attractive target for conquering tumor drug resistance problem [259-262]. However, few approaches were developed to show good abilities in overcoming lactic acidosis [263-267]. Meng et al. [268] developed novel ultrasound-responsive alkaline nanorobots. These nanorobots can autonomously accumulate at tumors via the enhanced permeability and retention (EPR) effect. With the ability to respond to external ultrasonic powering, it can destroy the acidic microenvironment of the tumor specifically, causing few adverse effects. As the first stimulus-responsive nanoscale therapeutic strategy for selectively relieving lactic acidosis, this nanorobot represents a promising approach for improving anti-tumor drug resistance efficacy (Fig. 6B).

### Nanorobots for conquering challenges in cancer treatments

Cancer cells exhibit uncontrolled growth, which invades or spreads to other parts of the body. Although chemotherapy, radiotherapy, photodynamic therapy, immunotherapy and many other therapeutic modalities are available choices for synergistic cancer therapies, it remains as a grand challenge for cancer treatment due to the diverse, complex, and heterogenic nature of tumors. Persistent efforts of scientists have been put forward to transport therapeutics to tumor mass through the introduction of nanomedicine, which was proven to be more effective and safer in face of conquering challenges in tumor treatments.

#### Nanorobots help resolve multidrug resistance problem

As a major reason responsible for the failure of cancer chemotherapy, multidrug resistance (MDR) is one of the most difficult challenges for cancer treatment [269]. The MDR phenomenon refers to the development of resistance of cancer cells, after repeated treatment, not only to the specific chemotherapeutic agent used, but also to other cytotoxic agents with different chemical structures or mechanisms of action, resulting in cross-resistance [270, 271]. MDR reduces the efficiency of treatment and makes the prognosis of cancer patients worse.

Cancer stem cells (CSCs) are closely associated with cancer recurrence and MDR occurrence [272]. However, CSCs are normally protected in niches inside the tumor bulk, promoting proliferation of cancer cells, and are difficult to target [273, 274]. With the EPR effect, nanocarriers can passively and selectively accumulate in the environment of tumors [275, 276]. Nanorobots, combined with CSC-targeting drugs, can selectively accumulate in solid tumors to effectively eradicate cancers [277].

Previously, Meng et al. recovered the lactic acidosis-mediated drug resistance by designing novel ultrasound-responsive alkaline nanorobots (AN-DSP) [268]. The nanorobots can rapidly release Na_2_CO_3_ to neutralize lactic acidosis in the TME with sensitive response to ultrasound stimulation. P-glycoproteins, the drug efflux pumps of cells, are overexpressed in tumor cells and could pump the anticancer compounds out of intracellular space, rendering the drug resistance of tumors [277, 278]. Nanoparticle formulations with various p-gp inhibitors will be one of the targets for nanorobots to prevent chemotherapy drugs being pumped out of the cancer cells. Polysiloxane nanosheets (PSX NSs) are approved by Fojtů M et al. that they have favorable properties for biomedical applications and are found to be highly effective in binding anticancer drugs [277]. Interestingly, polysiloxane nanosheets were found to be especially effective in the therapy of drug-resistant tumors, improving the effectiveness of up to 52%. They bound DOX on the surface of PSX NSs to become PSX@DOX. The PSX@DOX could reduce the growth of DOX-resistant tumors in vivo with 3.5 times better average efficiency than the free drug alone. The application of polysiloxane nanosheets to nanorobots will increase the therapeutic efficacy of anti-tumor drugs more specifically.

Numerous multifunctional nanocarriers, such as polymers mesoporous, silica nanoparticles and layered double hydroxide nanoparticles, were previously used to conquer MRD [279-281]. And co-delivery therapeutics through nanorobots will be one of the best approaches to achieve synergistic effects and eliminate hurdles of MDR in cancer treatments [282].

#### Nanorobots help resolve tumor hypoxia problem

**Fig. 7:**
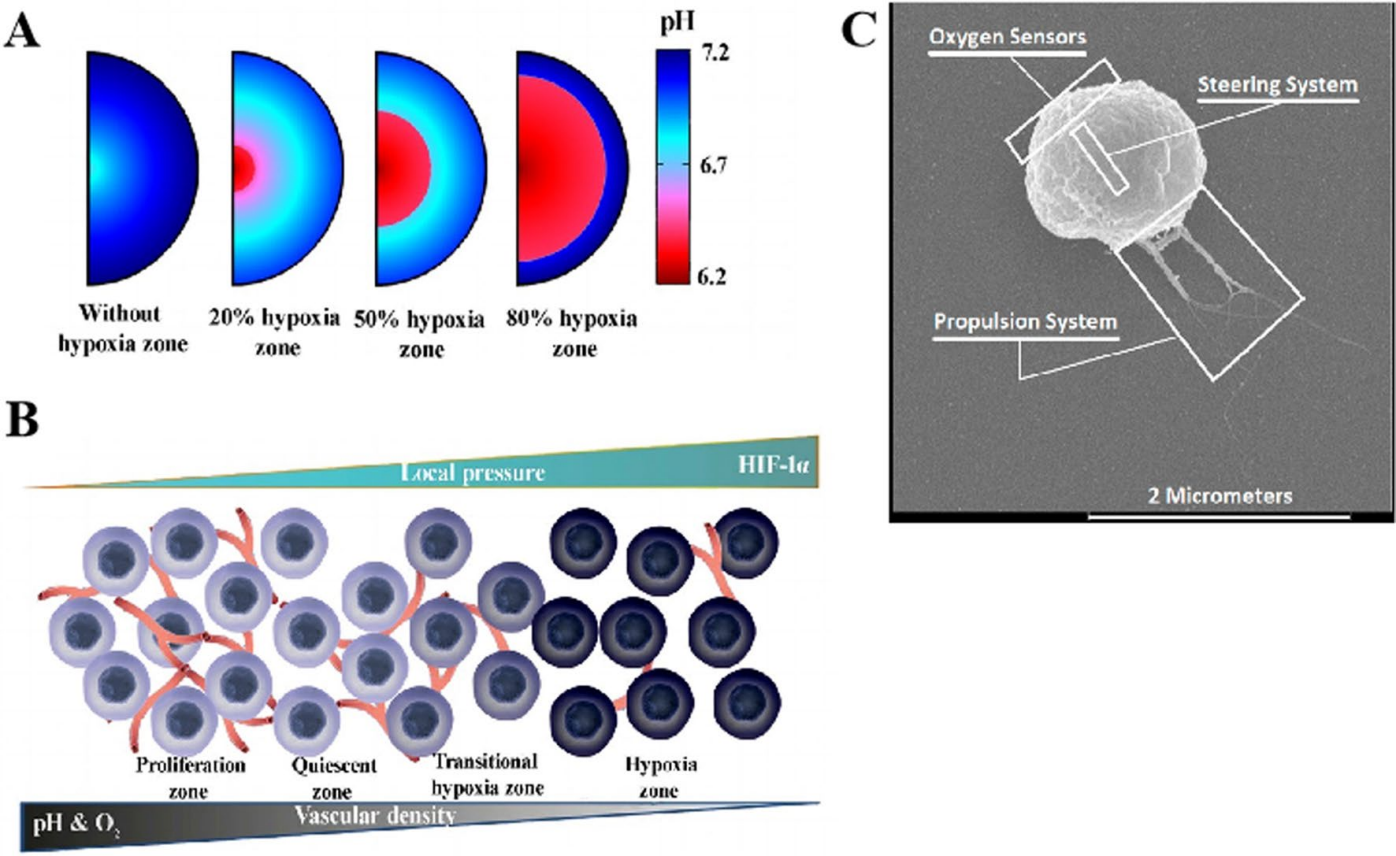
**A** Relationship between pH gradients and the extent of hypoxic regions in tumors. **B** Vascular densities and acidity levels in different regions of a tumor. Modified and reprinted from ref. [292]. Reproduced with permission, Copyright 2021, Springer Nature. **C** The main components of the MTB strain MC-1 nanorobot. Modified and reprinted from ref. [293]. Reproduced with permission, Copyright 2016, MDPI, Basel, Switzerland

As a salient pathological feature in TME, hypoxia was observed in 50–60% of solid tumors, influencing the cell cycle regulation, apoptosis evasion, stem cell maintenance, quiescence, and so on [283, 284]. Hypoxic adaptation holds a pivotal role in cellular energy metabolism, angiogenesis, trafficking and signaling, which results in difficulties to conventional cancer therapeutic approaches and enables cancer progression [285-287]. Acute hypoxia in tumor cells contributes to abnormal angiogenesis, which stimulates an aggressive, metastatic tumor phenotype, treatment-resistant tumor growth, thereby reducing overall patient survival [284, 288]. The remarkably diverse microenvironments in hypoxic tissues provide the potential doorway for suppressing the effects of nanomedicines via decreased oxygen partial pressure. Nanorobots display a new hope to the challenge of hypoxia-induced poor cancer therapy response [181, 289]. Reduced oxygen partial pressure is often treated as a stimulus for tumor-specific nanoparticles drug delivery. Hypoxia-induced factors 1 (HIF-1) and other sequences of HIFs play important roles for cancer cells to adapt to the hypoxic stress, leading to genetic transformations. The hypoxic regions of tumors can influence vascular density, and it means that regions with very low vascular density often have very low tissue oxygen levels. Based on the vascular density mathematical model, the extracellular and intracellular concentrations of a drug can be simulated by considering the binding/dissociation of the free drug from the cell surface receptors and the uptake by the cells. Acidic TME is another feature in the tumor hypoxia domains, which can be utilized as a stimulus for drug release in the tumor treatment (Fig. 7A) [290, 291]. M. Soltani et al. investigated a pH-responsive nanosized delivery system based on the extravascular release paradigm through a developed mathematical model (Fig. 7B) [292]. In order to develop the therapeutic nanorobots to be more effective in targeting hypoxic tumor regions, Martel S et al. [293] selected the magnetotactic bacteria (MTB) of the MC-1 strain with the ability to seek low oxygen concentration regions to serve as a candidate for implementing such a sophisticated cancer-fighting nanorobot (Fig. 7C). Following computer-based magnetotactic guidance to reach the tumor area, the self-propelled, sensory-based and drug-loaded nanorobot can be guided by a decreasing oxygen concentration toward the hypoxic regions. In this way, the MC-1 nanorobot can transport therapeutic payloads to the hypoxic zones of solid tumors following peritumoral injections.

## Nanorobots for precision surgery

Traditional surgery incorporates no nanosized surgical tools, which restricts surgical operation in such a nanoscale. Miniaturized nanorobots are small in sizes so that they could reach areas inaccessible to commonly used macro-sized surgical tools, such as catheters or blades. In addition, they may reduce the risk of infection and recovery period of time while improving surgical control and accuracy, and offering major advantages for high precision tumor-killing surgery. Currently, most previous studies in the field of robot-assisted surgery are microrobotic surgeries, which are of considerable promise in precision surgery and can overcome many of the above-mentioned limitations [294-298]. Most of untethered microscale robotic devices are in the centimeter-to-millimeter range. Further miniaturization of these miniature devices is also under development, such as microgrippers, microtraps, and microdriller, which were used for sample collection, tissue penetration, incision operations, etc. [299-304]. Reports about nanoscale robotic surgery for cancer treatment at a single cell level are still very rare. Some application scenarios and studies about nanorobots for precision surgery were summarized in Table 5*.* Disregarding the current developments of microrobots or nanorobots, there is still a long way to go on the road to precise surgery for cancer treatment. We believe that the potential of surgical nanorobots for tumor-killing will be greatly improved upon the development of multidisciplinary technologies, the choice of more advanced propulsion methods, real-time localization, and mapping with a more robust control system.

**Table 5:** Some application scenarios and studies about nanorobots for precision surgery

Surgery type	Author	Nanorobot name	Exploration details	Key highlights of nanorobot in surgery	Refs.
Biopsy/sample collection	Breger et al.	Self-folding thermo-magnetically responsive soft microgrippers	Using experiments and modeling, the authors designed, fabricated, and characterized photopatterned, self-folding functional microgrippers that combine a swellable, photo-cross-linked pNIPAM-AAc soft-hydrogel with a nonswellable and stiff segmented polymer (polypropylene fumarate, PPF) They also showed that we can embed iron oxide (Fe_2_O_3_) nanoparticles into the porous hydrogel layer, allowing the microgrippers to be responsive and remotely guided by external magnetic fields	Due to the low modulus of hydrogels such as poly(*N*-isopropylacrylamide-co-acrylic acid) (pNIPAM-AAc), they have limited gripping ability of relevance to surgical excision or robotic tasks such as pick-and-place. This nanorobot helps resolve the problem to some extent This study illustrated operation and functionality of these polymeric microgrippers for soft robotic and surgical applications	[299]
	Fernando Soto et al.	Multifunctional onion-like microtrap vehicle	The onion-inspired multi-layer structure contains a magnesium engine core and several outer chemoattractant and therapeutic layers Upon chemical propulsion, the magnesium core is depleted, resulting in a hollow structure that exposes the inner layers and serves as structural trap	The development of a multifunctional motile microtrap is capable of autonomously attracting, trapping and destroying of pathogens through controlled chemoattractant and therapeutic agent release Such a built-in chemical communication between synthetic microswimmers and motile microorganisms paves the way to diverse environmental and healthcare applications	[305]
	Fei Liu et al.	ExoTIC (exosome total isolation chip)	It is simple, easy-to-use, modular, which facilitates high-yield and high-purity EV (extracellular vesicle) isolation from biofluids	ExoTIC achieves an EV yield ~ 4–1000-fold higher than that with UC (ultracentrifugation), and EV-derived protein. microRNA levels are well-correlated between the two methods Authors utilized ExoTIC to isolate EVs from clinical cancer patient samples, including plasma, urine, and lavage, demonstrating the device’s broad applicability to cancers and other diseases	[306]
Tissue penetration	SaharJafari et al.	Magnetic particle drilling	Authors performed experiments with mouse cadavers that received 250 nm-wide intra-nasal magnetic rods intra-nasally under different combinations of external magnetic fields They found that the application of helical dynamic gradients to the particles (i.e., both rotational and linear) improved transport from the nose into the brain, as compared to linear magnetic gradients alone	This work illustrates the potential of using mechanical stimulation of neural tissue via unthreaded micro/nanorobots No particle track was observed in the brain after drilling, suggesting that the particles travel individually without causing brain damages, consistent with earlier studies in brain slices	[307]
	Juho Pokki et al.	cobalt–nickel microrobots	Coated and uncoated microrobots were investigated for their corrosion properties, and solutions containing coated and uncoated microrobots were tested for cytotoxicity by monitoring NIH3T3 cell viability In vivo tests inside rabbit eyes were performed using coated microrobots	The biocompatibility of micro-/nanorobot was enhanced by using a polypyrrole coating, presenting minimal inflammatory response in comparison with the noncoated counterpart Coated microrobots have the potential to facilitate a new generation of surgical treatments, diagnostics and drug delivery techniques, when implantation in the ocular posterior segment becomes possible	[308]
	Fernando Soto et al.	Acoustically triggered micro_x005fcannons (Mc)	Hollow conically shaped microcannon structures have been synthesized electrochemically and fully loaded with nanobullets made of silica or fluorescent micro_x005fspheres, and perfluorocarbon emulsions, embedded in a gel matrix stabilizer Applying a focused ultrasound pulse to the spontaneous vaporization of the perfluoro-carbon emulsions within the microcannon results in the rapid ejection of the nanobullets	The acoustic microcannons were able to deliver nanoparticles into phantom tissue, reaching a penetration length of ~ 20 µm This acoustic-microcannon approach could be translated into advanced microscale ballistic tools, capable of efficient loading and firing of multiple cargoes, offer improved accessibility to targeted locations and enhances tissue penetration properties	[309]
Intracellular delivery	Fernando Soto et al.	Nanoshell	Such shell-shaped nanomotors display highly efficient acoustic propulsion at the nanoscale by converting the ambient acoustic energy into mechanical motion Nanoshell motors exhibit a different propulsion behavior than that predicted by recent theoretical and experimental models for acoustically-propelled nanomotors	It was demonstrated the practical applications of the new nanoshell motors, including “on-the-move” capture and the transport of multiple cargoes and internalization and movement inside live MCF-7 cancer cells These structures provided higher cargo loading capacity as compared to previously described nanowires used for cellular internalization	[310]
	Xiangyu Jiao et al.	Janus nanocarrier (JNs)	Such a Janus nanocarrier with an Au surface could achieve efficient NIR propulsion, which are helpful for enhancing interactions with cells This nanocarrier could achieve deep tumor penetration by thermo-mechanical percolating cytomembranes and sequentially on–off controlled release	This system integrates the functions of active seeking, cytomembranes percolating, and on–off controlled release in one nanocarrier, which further improves the utilization of nanocarriers, and reduces the side effects of drugs These JNs achieve deep tumor penetration and are expected to reduce the side effects of nanocarriers and drugs	[311]
	Seungmin Lee et al.	Needle-type microrobot	The MRs were fabricated by 3D laser lithography and Ni/TiO_2_ layers were coated by physical vapor deposition The translational velocity of the MR is 714 μm/s at 20 mT and affixed to the target MT under the control of a rotating external magnetic field	Drug release from the paclitaxel (PTX)-loaded MR was characterized to determine the efficiency of targeted drug delivery This study demonstrated the utility of the proposed needle-type MR for targeted drug delivery to MT with various flow rates in vitro physiological fluidic environments	[312]
Biofilm degradation	Morgan M Stanton et al.	Magnetosopirrillum gryphiswalense (MSR-1)	MSR-1 can be integrated with drug-loaded mesoporous silica microtubes to build controllable micro-swimmers (biohybrids) capable of delivering antibiotics to target an infectious biofilm Under guidance of an external magnetic field controllable swimming power of the MSR-1 cells, the biohybrids are directed to and forcefully pushed into matured *Escherichia coli* (*E. coli*) biofilms	A bio-hybrid micro/nanorobot, composed of the integration of magnetotactic bacteria (MSR-1) with mesoporous silica loaded with ciprofloxacin (antibiotic), was explored to apply mechanical stress to *E. coli* biofilm Release of the antibiotic, ciprofloxacin, was triggered by the acidic microenvironment of the biofilm, ensuring an efficient drug delivery system	[313]
	Geelsu Hwang et al.	Catalytic antimicrobial robots (CARs)	CARs exploit iron oxide nanoparticles (NPs) with dual catalytic-magnetic functionality that 1) generates bactericidal free radicals, 2) breakdown the biofilm exopolysaccharide (EPS) matrix, and 3) remove the fragmented biofilm debris via an external magnetic field-driven robotic assembly	This robotic platform served to swept and remove biofilms over a flat surface, through a blocked capillary tube and to clean biofilms inside an interior tooth model These ‘kill-degrade-and-remove’ CARs systems could have significant impact in fighting persistent biofilm infections and in mitigating biofouling of medical devices and diverse surfaces	[314]

## Challenges faced by nanorobots for clinical cancer treatments

Nanorobotics hold great promise for revolutionizing cancer treatment by targeting cancer cells more precisely, reducing side effects, and improving treatment outcomes. However, numerous challenges remain to be addressed before nanorobots can be widely adopted for clinical cancer treatments [151, 315]. This section will discuss the technical complexity, precision, safety concerns, regulatory issues, funding and resources, as well as scalability challenges that currently hinder the development and implementation of nanorobotics in clinical cancer treatments.

### Technical complexity and precision

Designing and operating nanorobots for clinical cancer treatments involves overcoming multiple technical difficulties, such as developing nanoscale components, controlling their movements, and ensuring their stabilities. One major issue is the precise control of magnetic nanorobots through externally applied magnetic fields. The complexity of magnetic fields within narrow spaces and the interference from other electromagnetic waves make it difficult to achieve fine and precise control of nanorobots' motions, which may result in inaccurate targeting of tumor sites and potential harm to the human tissues/organs [316, 317].

In addition, the body fluid environment at low Reynolds numbers poses further challenges to the working accuracy and speeds of nanorobots. Interference from the biological environment may drastically affect the working accuracy and speeds of nanorobots [318]. Circulating proteins, blood cells and immune cells can interact with foreign particles leading to retardation in the movements and actions (or even removal) of nanorobots in blood [319, 320]. A higher power conversion efficiency is needed to promote the motions and actions of nanorobots in vivo. Different from blood, urine and saliva are two types of biological fluids that are also needed to be considered. Catalytic motors driven by diffusion and electrophoresis will suffer from reduced efficiency and metal corrosion in both cases.

### Safety concerns

The prospect of employing nanorobots in biomedical applications, particularly in cancer treatments, raises valid concerns regarding their safety and potential adverse effects on patients. Malfunctioning nanorobots could not only harm patients, but also lead to unintended side effects, further complicating the treatment process. For example, in cases of malignant tumors characterized by poorly developed blood vessels, the deployment of tubular DNA nanorobots containing thrombin may prove ineffective, ultimately failing to achieve the intended therapeutic goals [321, 322].

To address these concerns and ensure the safety and the efficacy of nanorobots for cancer treatments, a rigorous approach involving thorough preclinical and clinical testing is essential. Such an approach will help evaluate the biocompatibility, pharmacokinetics, and pharmacodynamics of these nanoscale devices in various biological environments. Additionally, implementing robust quality control measures during the manufacturing process will be crucial to minimize the risk of device failure and ensure consistent performance across different batches of nanorobots [323, 324].

### Regulatory issues

The current absence of comprehensive regulations governing the development and the use of nanorobots may hinder their widespread adoption by both public and private sector entities. Establishing appropriate regulatory frameworks that can effectively address the unique challenges posed by nanorobotics, while simultaneously promoting innovation and safeguarding public health, is essential for the successful integration of nanorobots into clinical cancer treatments [11].

Regulatory agencies should focus on developing guidelines that encompass the entire lifecycle of nanorobots, from design and development to clinical trials and post-market surveillance. Such guidelines should also include provisions for collaboration and data sharing among researchers, industry, and regulatory agencies to ensure that all stakeholders can contribute effectively to the development of safe and effective nanorobotic therapies [325].

### Funding and resources

Developing nanorobotics for clinical cancer treatments is an expensive endeavor that necessitates substantial funding and resources, as well as specialized equipments and human expertise. Securing adequate funding for research and development is paramount to advancing the field of nanorobotics and overcoming the various challenges it faces [326]. Government agencies, private organizations, and philanthropic institutions should collaborate to provide financial support for research initiatives and facilitate the translation of research findings into clinical applications.

Moreover, fostering collaboration among researchers, industry stakeholders, and regulatory agencies will be instrumental in expediting the development of nanorobotics for cancer treatments. Such collaborative efforts can help optimize resource allocation, drive innovation, and ensure that regulatory requirements are met, ultimately accelerating the translation of nanorobotic therapies from bench to bedside [325, 327].

### Scalability

Scaling up the development and production of large numbers of nanorobots for clinical cancer treatments is a formidable challenge due to the complex and time-consuming nature of the manufacturing processes. This scalability issue involves several key aspects that need to be addressed, including production techniques, cost, quality control, and supply chain management [328].

#### Production techniques

Traditional manufacturing techniques are often ill-suited for producing nanoscale devices. To mass-produce nanorobots, novel manufacturing methods and technologies must be developed that can reliably create intricate nanostructures with high precision. Advances in area such as self-assembly, 3D printing, and nanolithography may enable the production of nanorobots on a larger scale [329, 330]. Additionally, the integration of automation and machine learning into the manufacturing processes could further streamline production, reducing human errors and increasing overall efficiency.

#### Cost

The high cost of developing and manufacturing nanorobots is a significant barrier to their widespread adoption. Reducing the cost of production will require advances in nanomaterials, as well as improvements in manufacturing efficiency. For instance, the discovery of new, more cost-effective nanomaterials or the refinement of existing ones could help lower the overall cost of nanorobots. Moreover, increased collaboration between researchers, manufacturers, and funding agencies can foster the development of more affordable production techniques [331, 332].

#### Quality control

Maintaining high level of quality control during the mass production of nanorobots is essential to ensure their safety and efficacy. This entails developing robust testing and validation protocols to identify potential defects and ensure that nanorobots meet the necessary performance standards [333]. Implementing in-process monitoring and real-time feedback systems can further improve quality control by enabling manufacturers to identify and address issues more quickly during production.

#### Supply chain management

As the production of nanorobots increases, so too will be the complexity of their supply chain. Managing this complexity will require the development of efficient supply chain strategies that can handle the sourcing, storage, and transportation of nanoscale components and materials. This may involve the establishment of partnerships between manufacturers, suppliers, and logistics providers to optimize the flow of materials and ensure timely delivery of nanorobots to healthcare providers and patients [334, 335].

## Nanosubmarines in blood

As mentioned above, nanorobots are organic combinations of nanomechanical devices and molecular biological species (e.g., the combination of enzymes and nanogears) that can act as miniature doctors in biomedical engineering solving problems that are difficult for conventional medical devices to solve [19]. Such nanorobots can be injected into human blood vessels and become molecular robots operating in the vasculature, which could often be figuratively described as “nanosubmarine” [214]. These nanosubmarines derive energy from glucose and oxygen dissolved in the blood and are programmed by the physician to detect any object they encounter through external acoustic signals. Molecular nanosubmarines can perform whole-body health checkups, unblock blood clots in blood vessels, remove lipid deposits in the heart and arteries, engulf germs, kill cancer cells, and monitor lesions in the body, bringing a profound revolution in the diagnosis and treatment of modern medicine. Nanosubmarines can also be used to perform human organ repair work, such as repairing damaged organs and tissues, completing cosmetic surgery, and performing gene assembly work, i.e. removing wrong or harmful DNA from genes, or assembling normal DNA into chromosomes to make the organism function normally.

### Intelligent nanosubmarine to block tumor blood supply

With an in-depth understanding of the tumor microenvironment’s biological characteristics, including the tumor vascular microenvironment and the advancement of nanomedicine carrier construction technology, the development of nanosubmarine for cancer therapy has entered a new stage [336]. According to the pathological characteristics of different cancers, it is possible to design individualized functionally integrated intelligent drug delivery nanocarriers with precise preparation and controlled drug release for precise regulation of tumor microenvironment, which is also an important direction in the development of oncology nanomedicines. The development and applications of a new generation of functionally integrated and highly controllable smart drug carriers, such as mRNA nanosubmarine [337], DNA nanosubmarine based on DNA origami technology, provided new opportunities to realize nanomedicines to address the in vivo and intratumoral microenvironment in a more detailed manner [338]. In the field of tumor microenvironment regulation, a variety of molecules that are aberrantly expressed in the tumor vascular microenvironment could be used as specific targets for targeted drug delivery, and co-modification of one or more ligands on the surface of nanoparticles could facilitate the precise targeting of nanomedicines at specific sites of tumor vessels [339, 340]. In this section, we reviewed the important representative advances of smart nanosubmarines in anti-angiogenesis, vascular structure disruption and vascular embolization, and provide an outlook on the future development of smart nanosubmarines in this field, focusing on two strategies: tumor vascular blood supply blockage and tumor vascular modulation.

#### Anti-vascular nanosubmarine agents

Anti-vascular nanosubmarine agents (AVNAs) could be divided into angiogenesis inhibitors (AIs) and vascular-disrupting nanosubmarine agents (VDNAs) [341], of which angiogenesis inhibitors included sorafenib, avastin, apatinib mesylate, etc., which inhibited tumor angiogenesis by inhibiting the activity of angiogenic factors or receptors. VDNAs, such as sorafenib, bevacizumab, and lapatinib, inhibited tumor angiogenesis by inhibiting the activity of angiogenic factors or receptors, while VDNAs, such as Combretastatin A-4 (CA4) and its derivatives, induced secondary thrombosis by destroying the structures of existing blood vessels, both of which ultimately inhibited tumor growth and progression by blocking the blood supply [214]. However, most anti-vascular drugs are small molecules or monoclonal antibodies, which have inherent disadvantages such as poor water solubility, rapid clearance, and poor targeting. Their clinical efficacy and safety need to be improved by virtue of state-of-art nanotechnologies [341].

1. *Nanosubmarine with anti-vascular effects.* Certain types of nanomaterials used to construct nanomedicine carriers are inherently anti-vascular, and their use in anti-tumor drug delivery could synergistically enhance the anti-cancer efficacy of the drugs while simplifying the composition of nanomedicines and making them easier to prepare and apply. The anti-vascular effects of nanomaterials mainly include inhibition of angiogenesis and disruption of tumor vascular structure. Recent studies have found that gold, silver, copper oxide, cerium oxide, chitosan, and other nanoparticles have certain inhibitory effects on tumor angiogenesis, among which gold nanoparticles have been most intensively and widely studied [342]. Bhattacharya et al. reported that gold nanoparticles could inhibit the proliferation of human umbilical vein endothelial cells (HUVEC) induced by vascular endothelial growth factors (VEGFs) in vitro, suggesting a potential anti-angiogenic effect of gold nanoparticles [343]. They further found that gold nanoparticles could bind to the heparin-binding domain of VEGF and basic fibroblast growth factor (bFGF) in a size- and surface electrical-dependent manner, and competitively inhibit the binding of VEGF to the receptor VEGFR-2 to achieve the effect of inhibiting angiogenesis and tumor growth. In addition, Seo et al. further enhanced the vasopressor effect of gold nanoparticles by coupling VEGFR-1 antagonist peptide via sulfhydryl groups on the surface of gold nanoparticles, taking advantage of the easy functionalization of gold nanoparticles [344].

In addition to VDNAs that caused vascular disruption, nanosubmarines for photodynamic and photothermal therapy could also directly disrupt tumor vascular structures by absorbing light energy under near-infrared (NIR) light irradiation to generate reactive oxygen species (ROS). It was reported that a gadofullerene nanoparticles (GFNPs) could directly kill cancer cells by energy or electron transfer process under light illumination [345]. The GFNPs could act on tumor vascular endothelial cells, disrupting the endothelial junctions and thereby destroying the vascular structure. The anti-cancer effects of GFNPs in melanoma with rich blood supply were remarkable. Gao et al. used hollow copper sulfide nanoparticles loaded with ethylene azide and externally modified with peptides containing arginine-glycine-aspartate (Arg-Gly-Asp) sequence (RGD peptides) to prepare a “nano-bomb” targeting at tumor blood vessels [346]. Under the excitation of NIR light, the heat generated by copper sulfide led to the rapid production of large amounts of N_2_ gas, which blasts nearby tumor blood vessels and induced necrosis of surrounding cancer cells, resulting in complete regression of tumors in a single dose in a mouse subcutaneous transplantation cancer model without recurrence within 30 days’ follow-up, showing strong anti-cancer efficacy [346].

(2) *Nanosubmarine to improve anti-vascular efficacy.* A variety of nanosubmarine have been used for the delivery of anti-vascular drugs. Anti-vascular nanosubmarine exhibited strong stability, long circulation time, and exhibiting tumor-targeting ability, which ultimately manifested itself in the anti-cancer effects of increased efficiency and reduced toxicity. Zhang et al. used the internal porous structure of mesoporous silica nanoparticles (MSNs) to efficiently load bevacizumab, a VEGF antibody drug, and coupled cancer endothelial marker 1 (CEM1) monoclonal antibody on the surface of MSNs via a coupling reaction between the carboxyl group and the amide moiety in the antibody to achieve the tumor vascular targeting ability. The coupled CEM1 monoclonal antibody could specifically direct MSNs to the blood vessels of ovarian tumor sites, and significantly reduced the toxicities of MSNs to other normal tissues [347, 348]. Based on the local high level of glutathione (GSH) in the tumor tissues, Liu et al. synthesized a novel GSH-responsive polyethylene glycol (PEG)-based poly(alpha lipoic acid) (PALA) nanocarrier, to which CA4 was bonded ot the surface of the nanoparticles via the PEG chain [349, 350]. When PALA reached the tumor site, the high level of intra-tumor GSH reduced the disulfide bond in PALA, resulting in the degradation of the polymer and subsequent in situ release of CA4, which had strong anti-cancer activity without causing systemic toxicities. This study was the first to achieve tumor site-selective delivery of vascular-disrupting agents using simple nanosubmarines. Gao et al. designed a cationic lipid-coated poly(lactic acid)-hydroxyacetic acid copolymer (PLGA) nanosubmarine based on the phenomenon that sorafenib to conquer drug resistance in hepatocellular carcinoma by activating the stromal-derived cytokine-1α/C-X-C motif chemokine receptor 4 (SDF1α/CXCR4) axis [351]. The inner PLGA nanosubmarines carried sorafenib via hydrophobic interaction, and the outer cationic lipid layer could adsorb AMD3100 (CXCR4 antagonist) by electrostatic interaction, which impart the tumor site-targeting properties to the material while blocking CXCR4 activity. This nanosubmarine could deliver sorafenib specifically to the tumor site and restore the sensitivity of hepatocellular carcinoma cells to sorafenib, demonstrating the potential of CXCR4-targeted nanoparticles for clinical applications in delivering sorafenib specifically to the tumor site and overcoming acquired drug resistance in hepatocellular carcinoma [351].

#### Cancer vascular embolization nanosubmarine

In 1997, Huang et al. [352] employed a novel approach to deliver the extracellular region of tissue factor to tumor vascular sites using antibodies that targeted at the MHC II, with the goal of inducing tumor intravascular thrombosis and cutting off the tumor’s blood and nutrient supply, effectively “starving” the tumor tissue. This approach showed several key advantages over traditional cell-based anti-cancer therapies. Firstly, it was capable of inducing the death of a significant number of cancer cells in a tumor within a short time frame. Secondly, it did not require direct contact with the cancer cells, thus reducing issues associated with drug delivery to the tumor tissues. Finally, it was less likely to induce drug tolerance of cancer cells. Since then, various tumor-targeting nanomaterials have been developed to connect tissue factor extracellular regions and deliver various cytokines to the tumor site, such as L19, NGR, VEGF, chTNT-3, VCAM-1 and RGD. They have demonstrated promising therapeutic effects in animal models [353]. To date, the only cancer vasoembolic protein drug that has entered clinical trials is tTF-NGR [354]. This is likely due to the nonspecific embolism that occurs as a result of direct contact between the tissue factor fusion protein and blood after entering the body, which may cause serious toxicity. In order to address these limitations and safely deliver thrombin to the local tumor site, researchers are seeking new strategies that can intelligently identify the tumor vasculature and perform specific embolization.

1. *Smart DNA nanosubmarine loaded with thrombin.* In response to the non-druggable nature of thrombin and its transport dilemma, Li et al. developed a series of DNA nanorobots that could efficiently deliver thrombin to a tumor site, and release it in response to molecular signals from the tumor microenvironment [212]. These nanosubmarines were constructed from rectangular lamellar nanostructures using a single strand of M13 phage DNA as a template and multiple complementary short strands. Thrombin was immobilized on the upper surface of the nanostructures by modifying the DNA and hybridizing it with complementary DNA sequences. The nanosubmarines were designed with multiple pairs of “latch” structures on both sides that could be triggered to convolute the structure into a tubular form and encapsulate the thrombin inside. To ensure precise tumor vascular localization, the nanosubmarines were loaded with tumor-targeting nucleic acid adaptor AS1411 at both ends, which specifically targeted at nucleolin protein, a marker of tumor vascular endothelial cells [212]. When the DNA nanosubmarine reached the tumor vasculature, the double-stranded part of the "latch" sequence responded to the recognition of nucleolin and underwent a structural transformation, opening the nanosubmarine and exposing the thrombin, thus triggering the coagulation cascade and inducing thrombosis at the local tumor site. The results of protein and cellular level experiments showed that when the nucleophosmin was encountered by the nanosubmarine, it underwent structural deformation to expose the thrombin, leading to thrombus formation [212]. In vitro and in vivo stability studies demonstrated that the DNA nanosubmarines maintained their structural stability and thrombin activity quite well [212]. The nanosubmarines were successfully delivered to the tumor site in tumor-bearing rats, inducing thrombus formation in a variety of tumors including lung, ovarian, melanoma, and breast cancer. The application of DNA nanosubmarines for in vivo tumor embolization using non-druggable thrombin represents a major breakthrough in the field [212].
2. *Novel nanoembolic submarines.* Solid or liquid embolic agents used in clinical transarterial chemoembolization (TACE) often result in tumor revascularization or collateral circulation due to poor flow and absorption degradation [355, 356]. Temperature-sensitive polymeric nanogels with three-dimensional nano-networks have been used to solve the problem of existing tumor vascular embolization agents. The temperature-sensitive sol–gel phase transition allows the polymeric gels to flow freely through the catheter and respond to temperature changes after entering the bloodstream, forming a high-strength hydrogel network to embolize tumor vessels. The high drug loading capacity, controlled drug release, and the ability to integrate diagnostic reagents have further expanded the scope of the application of nanogels [357]. Several novel nano-embolic submarines that can block tumor vasculature in response to structural changes in the tumor microenvironment or specifically induce tumor vascular embolization have further broadened the idea of embolization therapy. Agemy et al. used the procoagulant effect of iron oxide nanoubmarines to design and synthesize a superparamagnetic iron oxide “nano-bug” coupled to two tumor-homing peptides (CRE KA and CRKDKC), which induced a broad coagulation response in the tumor vasculature without affecting normal blood vessels, effectively reducing tumor blood supply and inhibiting tumor growth [358]. Zhang et al. designed dual-responsive laminin mimic peptide (LMMP)-based nanosubmarines with both pH-responsive His6 sequence, tumor microthrombus-targeting peptide CREKA and fibril-forming sequence KLVFF, and the intravenously injected nanoparticles were enriched to the tumor site by CREKA. The His6 sequence responded to the acidic tumor microenvironment and thus underwent charge and molecular conformational changes, causing the laminin mimic peptide (LMMP) molecule to change from hydrophilic to hydrophobic, mimicking the process of fibril formation by natural laminin, forming a fibrous network in the tumor vasculature, blocking the tumor vasculature and inhibiting tumor growth [359]. Nanoembolic submarines mainly block tumor blood vessels through their structural changes, and do not cause the natural coagulation cascade reaction in the organism, so they have higher safety as compared with delivered coagulation factors, but also reduce the efficiency of local thrombosis and the permanence of embolization, how to balance their safety and efficacy needs to be further studied.

### Nanosubmarine for tumor vascular property modulation

Tumor vasculature is characterized by structural immaturity and high permeability, resulting in spatial and temporal heterogeneity of the blood flow in the tumor hypoxia domains, and increased interstitial fluid pressure, which are the main reasons for the ineffective enrichment of drugs in the tumor tissue during clinical treatment [360]. In addition, normalized tumor vasculature restores a certain degree of perfusion capacity, which increases drug delivery and thus enhances therapeutic efficacy [360, 361]. In addition, the enhanced permeability and retention (EPR) effect is the main way of nanomedicine accumulation in tumor sites. The EPR effect varies greatly among different tumor sizes and types [362]. Modulating the effect of EPR by regulating tumor vascular permeability to increase the penetration of nanomedicine has become one of the research focuses in oncology nanomedicine.

#### Tumor vascular permeability modulation by delivering nitrogen oxide (NO) donors

As a natural vasodilator, nitrogen oxide (NO) is one of the most commonly used regulators of tumor vascular permeability, and its donors increase blood flow by dilating blood vessels, thereby increasing the accumulation of drugs (especially nano drugs) in tumor tissues [361]. However, NO donors have a very short half-life and low stability and are prone to release NO in the physiological environment, leading to adverse reactions, largely limiting their clinical applications [363]. To address such problems, various nanosubmarines based on like liposomes, silicon dioxide, metal oxides, and polymeric nanoparticles have been used to deliver NO donors by controlling the behavior of nanocarriers to achieve spatial and temporal specific release of NO [363, 364]. NONOate is a commonly used NO donor that releases NO by protonation-induced self-decomposition under physiological conditions [365]. Tahara et al. used liposomes loaded with NONOate and enhanced its stability with an alkaline buffer inside the liposomes, while NONOate entering the acidic tumor microenvironment accelerated the decomposition of NO production and achieved the continuous release of NO in the tumor site without increasing NO in the blood, effectively dilating the tumor vasculature, and ultimately the accumulation of NONOate-laden liposomes in the tumor site was two times higher than that of empty liposomes [365]. The final accumulation of NONOate liposomes at the tumor site is two times higher than that of empty liposomes [365]. However, although the use of liposomes to encapsulate NO donors significantly enhances their stability. Liposome leakage may lead to the nonspecific release of NO from the blood environment due to the lack of stability of liposomes themselves, and the development of highly biocompatible and biodegradable polymeric carriers may expand the clinical application of NO donors in the future [366].

#### Normalization of tumor vasculature using nanosubmarine

Theoretically, normalization of tumor vasculature can be achieved by restoring the balance between pro- and anti-angiogenic signals, for example, by adding angiogenic inhibitors (e.g., endothelial inhibitors) or interfering with pro-angiogenic signals (e.g., angiogenic inhibitors) [367]. Li et al. used AuNPs to deliver rhES-AuNPs. rhES-AuNPs were passively accumulated at tumor sites through the EPR effects, prolonging the circulation time of rhES and increasing its aggregation at tumor sites. The combination with the chemotherapeutic agent 5-fluorouracil (5-FU) increased the delivery of 5-FU to a tumor site, showing a significantly stronger tumor suppressive effect than 5-FU monotherapy, and significantly prolonged the survival of mice [368]. As compared to free endothelial inhibitors, rhES-AuNPs have a longer action time to induce vascular normalization [368, 369]. Since existing drugs can only induce temporary tumor vascular normalization, this extended action time window is of great importance for clinical application. Therefore, the use of nanosubmarines to deliver vascular normalization inducers can not only overcome the inherent defects of drugs, but also facilitate the combination with other therapies. However, cancer vascularization may have different effects on nanoparticles that are themselves accumulated at the tumor site by EPR-dependent effects, i.e., improving the delivery of small size (around 10 nm in diameter) and hindering the delivery of larger size (around 100 nm in diameter or larger) nanoparticles [370]. Therefore, the size range of nanosubmarines needs to be carefully controlled when combining tumor vascularization strategies with other therapies.

## Directions and criteria for nanorobots in clinical cancer treatments

Technical reform of the current medical systems has attracted much attention from the nanomaterials and oncology societies. As the economy keeps growing, people are paying more attention to the healthcare. Many cancers are hard to diagnose at early stages, resulting in poor survival rates. More accurate and efficient medical diagnosis means are highly desired. Development of novel theranostic tumor-detecting or killing nanorobotic tools has become an important direction in the field of cancer treatments. Further clinical trials directly performed on cancer patients in the future can testify the efficacies of nanorobots in anti-tumor therapies. Current studies on tumor-detecting or killing nanorobots, however, are still in their infancy stage, and there is still a long way to go before being implemented in clinical practices. Significant future efforts are needed to promote nanorobots from animal model experiments to living human organisms. Without a doubt, many factors are essential to the development of nanorobots in clinical surgery of living bodies, including the advancement of safer materials with better biocompatibility and degradability, the advancement of higher power conversion efficiency, and other advancements in the key foundation of nanorobots.

In order to achieve widespread adoption in clinical practices, medical nanorobots designed for cancer treatments must meet several key criteria. The following prerequisites are the primary requirements that a medical nanorobot must fulfill:

1. *Medical safety assurance/requirements* ensuring the safety of medical nanorobots is of utmost importance for their successful integration into clinical practices. Rigorous preclinical and clinical testing must be conducted to evaluate the safety, biocompatibility, and potential side effects of nanorobots. Furthermore, the development of robust quality control measures during the manufacturing process is essential to minimize the risk of malfunctions and ensure consistent performance. Nanorobots should also be designed to be biodegradable or be easily removed from the body once their therapeutic purpose is completed, preventing long-term accumulation and potential toxicity.
2. *Biological mimicry.* Future nanorobots should emulate the natural intelligence of their biological counterparts, enabling precise control, high mobility, deformable structures, adaptive and sustainable operations, swarm-intelligent group behavior, complex functionality, and even self-evolutionary and self-replicating capabilities. This will allow for better adaptation to the human body and enhanced treatment efficacy.
3. *Swarm intelligence.* Advancing the swarm intelligence of nanorobots toward group motion planning, machine learning, and AI toolbox at the nanoscale is crucial for enhancing their capabilities in precision treatments [371-373]. This will enable coordinated actions and increased adaptability in complex biological environments.
4. *Integration with modern bioimaging and feedback control systems.* Future biomedical operation of nanorobots should be capable of coupling with modern bioimaging and feedback control systems for arbitrary four-dimensional navigation of many-nanorobot systems. This may enable the clustering and closed-loop feedback control of nanorobots within a living body, ensuring precise targeting and monitoring of treatment progress.
5. *Cutting-edge nanotechnologies.* New, innovative nanotechnologies are needed to propel nanorobots to the next level and enable cooperation with the latest advances in cancer medicine. For example, by attaching different types of biomolecules to nanorobots as guides, nanorobots can target specific cells, such as immune cells, to stimulate immune responses [18, 374-376]. This could inspire advances in cancer immunotherapy and further improve treatment outcomes.
6. *Financial feasibility.* In order to achieve lab-to-clinic transition, financial costs must be considered. Governments worldwide should support research into nanorobots for cancer treatments, and promote the further development of this novel medical robot nanotechnology. Simultaneously, costs should be controlled to favor the translation of advanced nanotechnology into the market and clinical use, making these treatments accessible to a broader patient population.

## Perspectives and conclusions

As described above, the development and application of nanorobots in cancer treatment are becoming a vigorous research area. To realize the full potential of nanorobots in the field of cancer treatment, material and AI scientists should work closely together with medical researchers for thorough investigations of the behaviors and functionalities of nanorobots, including drug delivery, targeted therapy, minimally invasive surgery, tumor detection and early diagnosis, and other advanced nanorobot-assisted comprehensive treatments. Considering the promising results achieved recently in both in vivo and in vitro experiments, scientists should look into the demands and the needs/challenges of oncology medical doctors, and design cancer-oriented medical nanorobots/nanosubmarines for specific diagnostic or therapeutic purposes to accelerate the translation of nanorobots/nanosubmarines cancer research to real-world clinical uses. We believe that using nanorobots as an integrated platform for multiple aims in different anticancer domains will soon be realized in the future.

The attainment of effective treatment for cancer through applications of nanomedicines necessitates the successful crossing of a vascular barrier of micron scale in order to exert therapeutic effects. Despite the high targeting efficiency demonstrated by actively targeted nanomedicines on cancer cells in vitro, the heterogeneous nature of the tumor microenvironment presents a challenge to their efficacies in vivo [336]. On the other hand, direct exposure of the vascular endothelial cells to the bloodstream offers convenient opportunities for targeted recognition and function of nanomedicines aimed at tumor blood vessels [377]. However, further optimization of the specificity of the relevant targets and tumor microenvironmental responses is imperative. The translation of experimental nanorobots/nanosubmarines into the clinical arena is limited by the complexity and heterogeneity of tumor biology, the lack of comprehensive understanding of nanomaterials-biology interactions, and the absence of scalable synthesis and mass production technologies for nanorobots/nanosubmarines [378]. The utilization of DNA nanotechnology in the form of DNA origami for thrombin delivery highlights the potential of precision drug delivery, yet substantial challenges such as immunogenicity, in vivo metabolic behavior, and large-scale production must be overcome before clinical implementation [379]. In the future, it is crucial to investigate the mechanisms of interactions between nanorobots/nanosubmarines and proteins/cells/tissues/organs in greater depths, regulate drug uptake through modulation of relevant target molecules, and prioritize the selection of nanomaterials with established biosafety and clear in vivo metabolic behavior. In addition, advanced preparation methods and characterization systems are imperative for the broadening of the clinical applications of nanorobots.

In the future, simple structured medical nanorobots are expected to evolute and become more sophisticated and capable of performing multiple medical functions and tasks, ultimately becoming true nanosubmarines in the bloodstream.

## Supplementary Information

**Additional file 1**: Confirmation of publication and licensing rights for Figure 1B

**Additional file 2**: Search results related to clinical trials of nanorobot on the website of www.clinicaltrials.gov.

## Data Availability

Not applicable (review article).

## Abbreviations

ATP: Adenosine triphosphate
CRISPR: Clustered regularly interspaced short palindromic repeats
DNA: Deoxyribonucleic acid
NIR: Near-infrared
PAM: Protospacer adjacent motif
sgRNA: Single-guide RNA
RNAi: RNA interference
TME: Tumor microenvironment
CNT: Carbon nanotubes
DOX: Doxorubicin
3D: Three-dimensional
PLGA: Poly(D, l-lactic-co–glycolic acid)
PDMS: Polydimethylsiloxane
Bi: Bismuth
SEM: Scanning electron microscopy
NEM: Nano-electrical–mechanical
NK: Natural killer
BBB: Brain–blood barrier
AIE: Aggregation-induced emission
MCDP: Multifocal cancer detection procedure
NGA: Niche genetic algorithm
MMO: Multimodal optimization
TJs: Tight junctions
DLGN: DNA logic-gated nanorobot
EASDs: Effector aptamer-tethered synergistic drugs
CAR-T: Chimeric antigen receptor T
TLS: Tumor lysis syndrome
RNase: Ribonuclease
EGFRs: Epidermal growth factor receptors
HApt: HER2-aptamer
tFNA: Tetrahedral framework nucleic acid
RBCs: Red blood cells
PLs: Platelets
EPR: Enhanced permeability and retention
MDR: Multidrug resistance
CSCs: Cancer stem cells
PSX NSs: Polysiloxane nanosheets
HIF-1: Hypoxia-induced factors 1
MTB: Magnetotactic bacteria
pNIPAM-AAc: Poly(Nisopropylacrylamide-co-acrylic acid)
PPF: Polypropylene fumarate
ExoTIC: Exosome total isolation chip
EV: Extracellular vesicle
UC: Ultracentrifugation
JNs: Janus nanocarrier
PTX: Paclitaxel
*E. coli*: *Escherichia coli*
CARs: Catalytic antimicrobial robots
NPs: Nanoparticles
EPS: Exopolysaccharide
AI: Artificial intelligence
PET: Positron emission topography
