# Supplementary material for: Advances of medical nanorobots for future cancer treatments
Source: J Hematol Oncol. 2023 Jul 14;16:74. doi: 10.1186/s13045-023-01463-z (PMC10347767; doi:10.1186/s13045-023-01463-z)
Supplement: Supplementary file 2 — Additional file 2: Search results related to clinical trials of nanorobot on the website of www.clinicaltrials.gov. [file 13045_2023_1463_MOESM2_ESM.doc]

| **Supplemental File 2**. Search Results Related to Clinical Trials of Nanorobot on the Website of [www.clinicaltrials.gov](http://www.clinicaltrials.gov/) | | | |
| --- | --- | --- | --- |
| **Search terms: Nanorobot** | | | |
| **Number** | **NCT Number** | **Drug/Device** | **Remarks** |
| 1 | NCT04644653 | Magnetic Swarming Nanorobots | For stroke treatment, not for cancer. |
| **Search terms: Nanocarrier, Tumor** | | | |
| **Number** | **NCT Number** | **Drug/Device** | **Remarks** |
| 1 | NCT03168061 | NC 6300 (Nanoparticle Epirubicin) |  |
| 2 | NCT02240238 | NC-6004 (Nanoparticle Cisplatin): Cisplatin-incorporating polymeric micelles |  |
| 3 | NCT00910741 | NC-6004 (Nanoparticle Cisplatin): Cisplatin-incorporating polymeric micelles |  |
| 4 | NCT03168035 | NC-4016: polymeric micellar nanoparticles incorporating 1,2-diaminocyclohexane platinum |  |
| 5 | NCT02043288 | NC-6004 (Nanoparticle Cisplatin): Cisplatin-incorporating polymeric micelles |  |
| 6 | NCT02817113 | NC-6004 (Nanoparticle Cisplatin): Cisplatin-incorporating polymeric micelles |  |
| 7 | NCT03771820 | NC-6004 (Nanoparticle Cisplatin): Cisplatin-incorporating polymeric micelles |  |
| 8 | NCT03109158 | NC-6004 (Nanoparticle Cisplatin): Cisplatin-incorporating polymeric micelles |  |
| **Search terms: Nano drug delivery, Tumor** | | | |
| **Number** | **NCT Number** | **Drug/Device** | **Remarks** |
| 1 | NCT02995603 | Nano-X patient rotation system |  |
| 2 | NCT04488224 | Nano-X Patient Rotation System | The Nano-X prototype is a horizontal patient rotation system that immobilises participants while being rotated on the horizontal axis. |
| 3 | NCT02367547 | Aminolevulinic Acid Nano Emulsion |  |
| 4 | NCT02367547 | Hexylaminolevulinate and Aminolevulinic Acid Nano Emulsion Versus Methylaminolevulinate |  |
| 5 | NCT02340858 | NanoKnife LEDC System |  |
| 6 | NCT05340725 | DEX-Nano-Rectal |  |
| 7 | NCT00672165 | Actinium-225-Labeled Humanized Anti-CD33 Monoclonal Antibody HuM195 | Targeted atomic nano-generators (Actinium-225-Labeled Humanized Anti-CD33 Monoclonal Antibody HuM195). |
| 8 | NCT01726894 | Irreversible Electroporation (Nanoknife®) |  |
| 9 | NCT01463709 | nanopulse |  |
| 10 |  | Electrosensing antibody probing system (e- Ab sensor) |  |
| 11 | NCT05456022 | Quercetin-encapsulated PLGA-PEG nanoparticles (Nano-QUT) |  |
| 12 | NCT04826913 | High Throughput Screening Device | High throughput screening device based on 3D nano-matrices and 3D tumors with functional vascularization. |
| 13 | NCT01442974 | Nab-paclitaxel | An albumin bound nano formulation of paclitaxel that targets SPARC, decreases tumor stroma density. |
| 14 | NCT02298608 | Nano Knife |  |
| 15 | NCT04918381 | CellFX System | Nano-Pulse Stimulation (NPS) |
| 16 | NCT03721042 | Olfactory measuring device | From a technical point of view, these are nano-sensors that mimic the olfactory receptors from the breath gas of the subjects. |
| 17 | NCT03417544 | Atezolizumab+Pertuzumab +Trastuzumab |  |
| 18 | NCT04310553 | Nano Knife |  |
| 19 | NCT02449135 | Digital subtraction angiography（DSA） |  |
| 20 | NCT04789486 | AGuIX | Gadolinium-based nanoparticles. |
| 21 | NCT02751606 | USPIO contrast agent (ferumoxtran-10) | In combination with Nano MRI on 7 Tesla to detect lymph node metastases. |
| 22 | NCT02751606 | Digital subtraction angiography（DSA） |  |
| 23 | NCT04554394 | CellFX Device | The CellFX System utilizes non-thermal, localized delivery of a timed series of low energy, nanosecond electrical pulses that can trigger regulated cell death. |
| 24 | NCT05331521 | Lomustine (CCNU) and Temozolomide |  |
| 25 | NCT04533919 | Nano-based analgesics |  |
| 26 | NCT03174275 | Carboplatin, Nab-Paclitaxel, Durvalumab | Nano-albumin bound paclitaxel. |
| 27 | NCT02079623 | NanoKnife |  |
| 28 | NCT04566029 | - | Evolution of proteomic profiles of intestinal microbiota in patients. |
| 29 | NCT02820454 | AGuIX | Gadolinium-based nanoparticles. |
| 30 | NCT03040453 | Microwave ablation |  |
| 31 | NCT03228095 | Novel nano-chemo sensors | Detection of volatile organic compounds in breath and headspace analysis. |
| 32 | NCT04738734 | CellFX System | THE CellFX System utilizes non-thermal, localized delivery of a timed series of low energy, nanosecond electrical pulses that can trigger regulated cell death. |
| 33 | NCT03661554 | BCMA Nano Antibody CAR-T Cells |  |
| 34 | NCT05700955 | Pembrolizumab and Temozolomide |  |
| 35 | NCT02022644 | Nanoliposomal irinotecan |  |
| 36 | NCT03854994 | Anti-CD19 CAR-T Cells Injection | Synthetic biology optimizing nano-vector T cells injection. |
